# Lymphocyte Activation Gene (LAG)-3 Is Associated With Mucosal Inflammation and Disease Activity in Ulcerative Colitis

**DOI:** 10.1093/ecco-jcc/jjaa054

**Published:** 2020-03-16

**Authors:** Stephanie M Slevin, Lucy C Garner, Conor Lahiff, Malcolm Tan, Lai Mun Wang, Helen Ferry, Borgel Greenaway, Kate Lynch, Alessandra Geremia, Stephen Hughes, Karen Leavens, David Krull, Daniel J B Marks, Katherine Nevin, Kevin Page, Naren Srinivasan, Ruth Tarzi, Paul Klenerman, Simon Travis, Carolina V Arancibia-Cárcamo, Satish Keshav

**Affiliations:** 1 NIHR Oxford Biomedical Research Centre, Translational Gastroenterology Unit, Oxford University Hospitals NHS Foundation Trust, Nuffield Department of Experimental Medicine, John Radcliffe Hospital, University of Oxford, UK; 2 Department of Laboratory Medicine, Changi General Hospital, SingHealth, Singapore; 3 Experimental Medicine Unit, GlaxoSmithKline, Gunnels Wood Road, Stevenage, UK; 4 GlaxoSmithKline, Collegeville, Pennsylvania, USA; 5 The Peter Medawar Building for Pathogen Research, University of Oxford, UK

**Keywords:** LAG-3, ulcerative colitis, immune checkpoint

## Abstract

**Background and Aims:**

Lymphocyte activation gene [LAG]-3 is an immune checkpoint and its expression identifies recently activated lymphocytes that may contribute to inflammation. We investigated the role of LAG-3 by analysing its expression and function in immune cells from blood and tissue of patients with ulcerative colitis [UC].

**Methods:**

The phenotypic properties of LAG-3^+^ T cells were determined by flow cytometry, qRT-PCR and single-cell RNA-sequencing. LAG-3^+^ cells were quantified and correlated with disease activity. The functional effects of LAG-3^+^ cells were tested using a depleting anti-LAG-3 monoclonal antibody [mAb] in a mixed lymphocyte reaction [MLR].

**Results:**

LAG-3^+^ cells in the blood were negligible. LAG-3^+^ lymphocytes were markedly increased in inflamed mucosal tissue and both frequencies of LAG-3^+^ T cells and transcript levels of *LAG3* correlated with endoscopic severity. LAG-3 expression was predominantly on effector memory T cells, and single-cell RNA-sequencing revealed *LAG3* expression in activated and cytokine-producing T cell subsets. Foxp3^+^CD25^hi^ Tregs also expressed LAG-3, although most mucosal Tregs were LAG-3^−^. Mucosal LAG-3^+^ cells produced mainly interferon γ [IFNγ] and interleukin-17A. LAG-3^+^ cell numbers decreased in patients who responded to biologics, and remained elevated in non-responders. Treatment with a depleting anti-LAG-3 mAb led to a reduction in proliferation and IFNγ production in an MLR.

**Conclusions:**

LAG-3^+^ cells are increased in the inflamed mucosa, predominantly on effector memory T cells with an activated phenotype and their cell numbers positively correlate with disease activity. Depleting LAG-3 eliminates activated proliferating T cells, and hence LAG-3 could be a therapeutic target in UC.

## 1. Introduction

Ulcerative colitis [UC] is a chronic mucosal inflammatory condition in which T lymphocytes play a critical role.^1,2^ The importance of T cells is demonstrated both by murine T cell transfer models of experimental colitis,^3^ and by the clinical efficacy of drugs targeting T cell trafficking in UC.^4–6^ Selective depletion of newly activated T cells, with sparing of resting lymphocytes, could represent a promising therapeutic strategy in this context. Therefore, strategies to limit the trafficking of lymphocytes from the circulation to the tissues could be augmented or substituted by treatments that specifically deplete activated T cells when they encounter a trigger from the T cell receptor.

Lymphocyte activation gene [LAG]-3 is a transmembrane receptor that is upregulated on activated CD4^+^ and CD8^+^ T cells, as well as on a subset of natural killer [NK] cells.^7^ Composed of four extracellular immunoglobulin domains, LAG-3 is structurally similar to the CD4 co-receptor and, like CD4, binds to MHC Class II.^8^ It functions as an inhibitory co-receptor, comparable to programmed cell death protein [PD]-1 and cytotoxic T lymphocyte antigen [CTLA]-4.^9^ Upon ligation of a stable complex of peptide and MHC Class II,^10^ LAG-3 mediates a cell-intrinsic negative inhibitory signal via two distinct mechanisms that are dependent on the FXXL motif in the membrane-proximal region and the C-terminal EX repeat.^11^

Blocking LAG-3 and PD-1 signalling has been shown to have synergistic effects against tumoral immune escape,^12,13^ and many clinical trials investigating combinatory blockade are ongoing. Conversely, a deficiency in checkpoint inhibitor signalling leads to the development of immune-mediated inflammatory diseases [IMID] as illustrated by CTLA-4 and PD-1 knockout mice.^14–16^ In line with this, some patients with advanced cancer treated with immune checkpoint inhibitors develop induced IMID in certain organs, which can provoke inflammation, including colitis.^17^ Achieving selective immunosuppression of activated T cells by targeting such co-inhibitory receptors could clinically be effective in IMID.

The knockout mice and the newly identified IMID in oncology have set the stage for LAG-3 and PD-1 being important and useful as they define: tumour-specific effector T cell subsets at the tumour site,^18^ exhausted T cells in chronic infections^19,20^ and, in our case, recently activated T cells in IMID such as UC. In support of this proposition, antibodies that deplete LAG-3^+^ lymphocytes prolong transplant survival in a rat cardiac allograft model,^21^ and abrogate Th1-driven inflammation in a delayed-type hypersensitivity challenge in non-human primates.^22^ Recently, single-cell RNA-sequencing of ileal biopsies from patients with Crohn’s disease found upregulation of LAG-3 expression in the cytotoxic T lymphocytes of the intestinal mucosa.^23^

Here we show that LAG-3 expression is elevated in the inflamed colonic mucosa from patients with active UC, and correlates with endoscopic and histological disease activity. It is principally expressed on T cells with an effector memory phenotype including heterogeneous populations of activated CD4^+^ T cells expressing of T helper-associated cytokines and granzyme-producing cytotoxic CD8^+^ T cells. *Ex vivo*, stimulated LAG-3^+^ colonic T cells are capable of robust production of interferon γ [IFNγ] and interleukin [IL]-17A. Furthermore, LAG-3^+^ cell numbers decline following successful treatment of UC with anti-tumour necrosis factor [anti-TNF] therapy or vedolizumab and remain elevated in non-responders, validating this as a viable target for novel therapies, including patients refractory to existing medications.

## 2. Materials and Methods

### 2.1. Study subjects

Intestinal biopsy and blood samples were collected from patients [diagnosed by standard criteria]^24,25^ attending the John Radcliffe Hospital, Oxford, UK [prospective cohort]. In some cases, biopsies were taken from both inflamed and uninflamed bowel in patients with UC. Non-inflammatory bowel disease [IBD] controls are patients who undergo colonoscopy due to gastrointestinal symptoms [diarrhoea, bloody stools and abdominal cramps] but are not diagnosed with IBD or show any signs of mucosal inflammation. Additional colonic tissue samples for immunohistochemistry [IHC] were retrieved from the Oxford Gastrointestinal Illness [GI] cohort and biobank. Healthy volunteers for blood donation were identified through the Translational Gastroenterology Unit at the University of Oxford. Details of participants included in experiments are provided in Table 1.

**Table 1.** Demographic and clinical characteristics of the groups of study subjects [prospective cohort]

	Non-IBD controls*	UC uninflamed	UC inflamed†
Number	9	8	34
Age, median [range], years	41 [21–72]	31 [20–49]	40 [14–75]
M/F	5/4	5/3	19/15
**Disease extent [%]**			
E1 Proctitis	N/A	0 [0]	2 [6]
E2 Left-sided	N/A	2 [25]	17 [50]
E3 Extensive	N/A	6 [75]	15 [44]
PSC	0	3 [38]	5 [15]
Disease duration, median [range], years	N/A	8 [0–16]	7 [0–46]
UCEIS, median [range]	N/A	0 [0–2^#^]	4 [1–7]
Nancy Score, median [range]	N/A	0 [0–3^#^]	3 [0–4]
**Medication at endoscopy**			
Thiopurines [%]	N/A	4 [50]	12 [35]
5-ASA [%]	N/A	6 [75]	23 [68]
Corticosteroids [%]	N/A	1 [13]	9 [26]
Infliximab [%]	N/A	1 [13]	1 [3]
-Weeks	N/A	58	8
Adalimumab [%]	—	0 [0]	3 [9]
-Weeks, median [range]		—	48 [8–78]
Vedolizumab [%]	N/A	0 [0]	5 [15]
-Weeks, median [range]	—	—	24 [13–39]

Abbreviations: IBD, inflammatory bowel disease; UC, ulcerative colitis; PSC, primary schlerosing cholangitis; UCEIS, ulcerative colitis endoscopic index of severity; 5-ASA, 5-aminosalicylic acid; N/A, not applicable.

*Non-IBD controls are patients deemed healthy during endoscopy.

†Sixteen are paired samples [uninflamed and inflamed biopsies from the same patient].

^#^Uninflamed tissue was taken from a patient with colonic inflammation at a different location. Biopsies were from areas with no macroscopic inflammation and confirmed to be uninflamed on histology.

Biobanked colonic tissue samples were retrieved from patients with active disease, before and after treatment with either anti-TNF [infliximab] or anti-integrin [vedolizumab] therapy [historical cohort] Table 2. Patients were classified as responders to therapy based on endoscopic improvement, defined as a reduction of the ulcerative colitis endoscopic index of severity [UCEIS]^26^ score of ≥ 3, and on histological improvement, defined as a reduction in the Nancy^27^ score of ≥ 2 points. Ethical approval for the study was provided by the National Research Ethics Committees of the UK National Health Service [NHS] under reference numbers 09/H0606/5, 11/YH/0020 and 16/YH/0247. Colonic biopsies for immunofluorescence for LAG-3 were sourced through an external vendor, Tissue Solutions, with appropriate ethical approval for research. The human samples used in this publication were ethically sourced and their research use was in agreement with the informed consent.

**Table 2.** Historical cohort categorised by response to biological therapy

	Responders	Non-responders	*p* value
Number	11	12	—
Age; median [range], years	29 [19–54]	26 [18–52]	0.11
Gender [M/F]	6/5	5/7	0.68
**Disease extent [%]**			
E1 Proctitis	1 [9]	0 [0]	
E2 Left-sided	1 [9]	5 [42]	
E3 Extensive	9 [82]	7 [58]	0.14
Disease duration; median [range], years	3 [1–10]	3 [1–8]	0.48
UCEIS^**#**^	5 [3–7]	5 [3–6]	0.11
Nancy score^#^*	3 [2–4]	4 [3–4]	0.03
**Medication**			
Infliximab	3	4	
Vedolizumab	8	8	0.75
IHC scores [IQR]			
LAG-3 [total]	4.6 [0.8–14]	4 [0.8–10.4]	0.77
iMC [mean]	53 [39–80]	66 [40–86.7]	0.16

Abbreviations: UCEIS = Ulcerative Colitis Endoscopic Index of Severity; CRP = C-reactive protein; IHC = immunohistochemistry; IQR = interquartile range; iMC = intra-mucosal calprotectin.

^#^UCEIS and Nancy scores are from baseline.

*Modified Riley score was recorded in two patients and equivalent Nancy values were imputed.

### 2.2. Cell isolation

Peripheral blood mononuclear cells [PBMCs] were isolated using Lymphoprep [STEMCELL Technologies] density gradient centrifugation; red blood cells [RBCs] were lysed using 1× RBC lysis buffer [eBioscience] and resuspended in FACS buffer (phosphate-buffered saline [PBS] with 1% bovine serum albumin [BSA] and 2 mM EDTA [Sigma Aldrich]).

Lamina propria mononuclear cells [LPMCs] from the colon were isolated from up to seven pairs of pinch biopsies taken from inflamed and uninflamed areas of the colon at endoscopy. Biopsies were collected in complete [c]-RPMI supplemented with 10% fetal calf serum [FCS, Sigma Aldrich], 1% penicillin/streptomycin and 1% l-glutamine [all from Invitrogen]. Biopsies were transferred to gentleMACS C tubes [Miltenyi Biotec] containing c-RPMI with 1 mg/mL Collagenase D [Sigma-Aldrich] and 0.01 mg/mL DNase [Roche] and agitated for 30 s on the gentleMACS dissociator [Miltenyi Biotec] using the ‘gentle’ setting. Thereafter, biopsies were incubated for 1 h in a shaking incubator at 37°C, and then agitated for 30 s on the gentleMACs dissociator using the ‘vigorous’ setting. The dissociated biopsy material in c-RPMI was then strained through a 0.77-µm filter to collect the cells released from the tissue.

### 2.3. Flow cytometry

Cells were stained with the following antibodies: CD4-FITC, integrin β7-FITC, LAG-3-PE, CD3-PE-CF594, CD25-PE-Cy7, CCR9-PE-Cy7, CCR7-APC, CD161-APC, Fixable Viability Dye-eFluor-780, γδTCR-BV421, TCRvα7.2-BV421, CD45RA-BV510, CCR6-BV605, CD8a-BV650, CXCR3-BV711, CD103-BV711 and CD127-BV785 [Supplementary Table 1A]. Samples were acquired on a FACS LSRII Special Order Research Product [SORP] and FACS Aria III [BD]. Data were analysed with FlowJo V10 [Tree Star].

### 2.4. RNA extraction and qRT-PCR

Intestinal biopsies were stored in RNA Later [Qiagen] at −80°C. Tissue was homogenized in a tube containing glass beads in RLT buffer [Qiagen] plus β-mercaptoethanol [Sigma Aldrich] using the FastPrep 24 instrument [MPBio]. RNA was isolated using the RNeasy Mini kit [Qiagen] including the Qiashredder and DNase steps. The RNA was analysed by Epistem and the following was performed. RNA concentration was quantified by measuring UV absorbance at 260 nm using a NanoDrop 8000 spectrophotometer [ThermoFisher Scientific] and RNA quality was assessed using RNA 6000 Nano chips on an Agilent 2100 Bioanalyser [Agilent Technologies]. A minimum RNA integrity number [RIN] of 6.5 was required for inclusion in subsequent analysis. All samples were screened by quantitative real-time PCR [qRT-PCR] against a panel of TaqMan Gene Expression Assays [ThermoFisher Scientific] comprising genes of interest and two housekeeping genes [*POLR2G* and *POLR2J*], using the Fluidigm BioMark HD microfluidic PCR platform as described in the Fluidigm Gene Expression TaqMan Workflow [Supplementary Table 1B]. A normalized input [31 ng] of RNA from each sample was converted to cDNA using Reverse Transcription Master Mix [Fluidigm]. TaqMan PreAmp Master Mix [ThermoFisher Scientific] and pooled TaqMan assays were used for 20 cycles to pre-amplify specific transcripts and qPCR was performed in duplicate on a 1:8 dilution of amplified cDNA using BioMark 48.48 Dynamic Arrays and the BioMark HD instrument [Fluidigm]. Ct values were calculated by the Fluidigm Real-Time PCR Analysis software version 4.1.3.

### 2.5. Intracellular cytokine analysis

Cells were stimulated with either: phorbol myristate acetate [PMA; 50 ng/mL or 100 ng/mL, Sigma Aldrich] and ionomycin [1 µg/mL; Sigma Aldrich] for 2-4hrs, soluble anti-human CD3 [1 µg/mL, UCHT1] and soluble anti-human CD28 [1 µg/mL, CD28.2, both BD Biosciences] overnight, or anti-human CD3 and recombinant human IL-12 [1ng/mL, Life Technologies] overnight. GolgiStop and GolgiPlug [both BD Biosciences] were added at the beginning of the PMA/ionomycin stimulation, or 4 h before the end of stimulation for the CD3/CD28 or CD3/IL-12 stimulation. Cells were stained with fixable viability dye and fixed for 1 h [Foxp3 staining buffer set, eBioscience]. Cells were then washed twice with 1× permeabilization buffer and stained for all relevant surface and intracellular markers for 1 h. The antibodies used were CD4-FITC, CD8-BV650, CD45-AF700, Fixable Viability Dye-eFluor-780, GM-CSF-PE-Dazzle, CD25-BV786, IL-10-PE-Cy7, IFNγ-PE-Dazzle, IL-22-PE-Cy7, IL-4-BV711 and IL-17A-eFluor450 [Supplementary Table 1A].

### 2.6. Transcription factor staining

Foxp3 expression was analysed using the Foxp3 staining buffer set [eBioscience]. Cells were stained with fixable viability dye eFluor-780, then fixed using the fixation buffer for 1 h. Cells were washed twice with 1× permeabilization buffer and stained for Foxp3 and relevant surface markers for 1 h [Supplementary Table 1A]. The antibodies used were CD4-FITC, CD8-BV650, CD45-AF700, Fixable Viability Dye-eFluor-780, CD25-BV786, LAG-3-PE and Foxp3-BV421.

### 2.7. Single-cell data generation and analysis

#### 2.7.1. Library preparation and sequencing

Colonic LPMCs were isolated from five pairs of pinch biopsies from four patients with active UC [three distal colon samples, one ascending colon sample; Supplementary Table 2] as described above. Dead cells were excluded using the Dead Cell Removal Kit [Miltenyi Biotec] and CD3^+^ T cells were positively selected using CD3 MicroBeads [Miltenyi Biotec] following the manufacturer’s instructions. Purified cells were cryopreserved in a 90% fetal calf serum [FCS]/10% dimethyl sulfoxide [DMSO] solution in liquid nitrogen until ready for use. Combined single-cell RNA-sequencing and single-cell T cell receptor [TCR]-sequencing libraries were generated using the 10x Genomics Chromium Single Cell V[D]J Reagent Kits [v1.0 Chemistry] following manufacturer’s instructions [CG000086 Rev J]. For each sample, approximately 16 500 cells were loaded per channel of the Chromium Controller, capturing an average of 5456 cells [range: 4487–6645]. The quality and concentration of final libraries were assessed using an Agilent TapeStation and a Qubit 2.0 Fluorometer [Thermo Fisher Scientific]. Libraries were sequenced on an Illumina HiSeq 4000 to a mean depth of 63 000 reads per cell [range: 56 000–68 000] for gene expression libraries and 22 089 reads per cell [range: 15 199–27 244] for TCR libraries. Cell capture, library preparation and sequencing were performed at the Oxford Genomics Centre [Wellcome Centre for Human Genetics, University of Oxford].

#### 2.7.2. Data pre-processing

Unique molecular identifier [UMI] count matrices [gene expression], and single-cell V[D]J sequences and annotations, were generated using 10x Genomics Cell Ranger software [v3.0.1 or v3.0.2]. For gene expression data, FASTQ files were generated using cellranger mkfastq. Reads were aligned to the 10x Genomics human GRCh38 reference genome [v3.0.0] and quantified using cellranger counts. The four samples were aggregated using cellranger aggr with normalization for read depth [number of reads confidently mapped to the transcriptome]. For TCR data, FASTQ files were generated using cellranger mkfastq, and TCRs were assembled and annotated using cellranger vdj with the 10x Genomics human GRCh38 V[D]J reference [v3.0.2].

#### 2.7.3. Quality control

Using the Seurat R package [v3.0.1],^28^ the aggregated raw gene expression matrix was filtered to retain only genes expressed in at least ten cells and only cells expressing at least 200 genes. Cells from the ascending colon sample were removed for most analyses, as described in the results. All T cell receptor [TCR] and B cell receptor [BCR] genes were removed, so that transcriptional clustering was not influenced by the expression of TCR or BCR gene segments. Next, valid cell barcodes were defined based on the UMI frequency distribution. The lower UMI threshold, intended to remove dead cells and cell-free RNA, was set at the local minimum of the UMI distribution to the left of the mode UMI count. The upper UMI threshold was determined individually for each sample using a novel approach that made use of the matched TCR-sequencing data [see below]. Appropriate setting of the upper UMI threshold is important to retain as many viable cells as possible, whilst removing cell multiplets [predominantly doublets]. Further cell filtering was performed to remove outliers that were potentially dead cells or multiplets [cells with ≥ 10% mitochondrial reads and/or ≥ 3000 genes were removed].

#### 2.7.4. Novel approach to determine the upper UMI threshold

The majority of T cells express a single TCRα chain and a single TCRβ chain. However, as thymic rearrangement of TCRα gene segments occurs simultaneously on both human chromosomes, a subset [10–19%] of T cells express two productive TCRα chains.^29–32^ A smaller fraction [6–7%] of T cells express two TCRβ chains,^30–32^ due to ‘leakiness’ in allelic exclusion at the TCRβ locus. Expression of three TCRα and/or three TCRβ chains is, however, impossible for a diploid cell. Therefore, any T cells for which more than two TCRα and/or two TCRβ chains are detected by single-cell TCR-sequencing must be multiplets. This knowledge was used to determine a suitable upper UMI threshold.

Cells were divided into groups depending on whether they had zero, one, two or three TCRα chains. As expected, compared with cells expressing a single TCRα chain [which were presumed to be predominantly live single cells], UMI counts were significantly decreased and increased, respectively, in cells with zero or three TCRα chains [Supplementary Figure 1A]. Moreover, the percentage of mitochondrial reads in cells with zero TCRα chains was significantly increased relative to cells with a single TCRα chain [Supplementary Figure 1B], confirming enrichment of dead cells amongst cells lacking TCRα chains. Analogous results were obtained for cells grouped by TCRβ chain frequency [Supplementary Figure 1C, D].

For each sample, the upper UMI threshold was set at the 85^th^ percentile of the UMI distribution for all cells with one TCRα chain and/or one TCRβ chain [Supplementary Figure 1E]. This cut-off was selected as it removed most cells with greater than two TCRα and/or two TCRβ chains [which must be multiplets], while retaining the majority of cells with a single TCRα and/or TCRβ chain [Supplementary Figure 1F, G]. With different datasets, the appropriate percentile at which to set the upper UMI threshold may vary. For the aggregated analysis of samples, the mean of the individual sample thresholds was used.

#### 2.7.5. Normalization, dimensionality reduction and clustering

Downstream analysis steps were performed using the Seurat R package [v3.0.1],^28^ as described in the tutorials [https://satijalab.org/seurat/]. Briefly, data were normalized and variable genes were identified using sctransform,^33^ with mitochondrial read fraction regressed out. Dimensionality reduction was performed using principal component analysis [PCA] with the variable genes as input. The top 30 principal components were used as input for graph-based clustering [resolution 0.8] and for dimensionality reduction using uniform manifold approximation and projection [UMAP].^34^ Outlier non-CD3^+^ T cell clusters for which the majority of cells lacked *CD3E* expression were removed, and the analysis steps were repeated, including sctransform normalization and variable gene selection, dimensionality reduction and clustering. For the separate analyses of CD4^+^ T cells and CD8^+^ T cells, the data were split into subsets to retain only the desired clusters and the analysis steps were repeated.

#### 2.7.6. Differential expression analysis

Differentially expressed genes between each cluster and all other cells were identified using the FindAllMarkers function with default parameters [Wilcoxon Rank Sum test, log fold-change ≥ 0.25]. Differentially expressed genes were filtered to keep only those with an adjusted *p*-value [based on Bonferroni correction using all features in the dataset] < 0.05. Clusters were labelled according to their key marker genes, or annotated as known T cell subsets based on the literature.

#### 2.7.7. Pathway enrichment analysis

Pathway enrichment analysis was performed using the ReactomePA R package.^35^

### 2.8. Cytokine secretion assays

LPMCs were isolated from the inflamed colonic biopsies of patients with UC [*n* = 6] as described above. Cells were stained for IFNγ, IL-17A and IL-10 using the Cytokine Secretion assay [Miltenyi Biotec], according to the manufacturer’s instructions. In brief, the cells were stimulated for 2 h with PMA [50 ng/mL] and ionomycin [1 µg/mL] to induce cytokine production. Thereafter, the cells were incubated with a cytokine catch reagent for 5 min on ice and then incubated for 45 min at 37°C to allow cytokine secretion. Secreted cytokines bind to the corresponding catch reagent on the surface of secreting cells. These cells were then incubated with the cytokine detection antibody conjugated for 10 min on ice to either FITC or APC for detection by flow cytometry.

### 2.9. Sorting LAG-3^+^ cells

Freshly isolated PBMCs from non-IBD controls were stimulated overnight with soluble anti-human CD3 [1 µg/mL] and soluble anti-human CD28 [1 µg/mL, both BD Biosciences] to induce LAG-3 surface expression. Cells were stained with the following antibodies: CD4-FITC, CD8a-BV650, CD45-AF700, LAG-3-PE, and 4’,6-Diamidino-2-Phenylindole, Dilactate [DAPI, Biolegend, Supplementary Table 1A]. The PBMCs were sorted on a FACSAriaIII using a 70-µm nozzle and those T cells [CD4^+^ and CD8^+^] that were LAG-3^+^ or LAG-3^−^ were sorted into separate collection tubes.

To determine cytokine expression, sorted LAG-3^+^ and LAG-3^−^ cells were cultured for 3 h at 37°C in modified Dulbecco’s medium [MDM, Life Technologies] with 10% FCS and 25 mM HEPES [Life Technologies] with or without PMA [100 ng/mL]/ionomycin [1 µg/mL] to induce cytokine production and with GolgiPlug and GolgiStop [BD Biosciences] to prevent extracellular secretion of cytokines. Cells were stained with fixable viability dye eFluor-780 [eBioscience], then fixed using the fixation buffer for 1 h. The cells were washed twice with 1× permeabilization buffer and stained with the following antibodies: CD4-FITC, CD8-BV650, CD45-AF700, LAG-3-PE, GM-CSF-PE-Dazzle, Foxp3-BV421, CD25-BV786, IL-10-PE-Cy7, IFNγ-PE-Dazzle, IL-22-PE-Cy7, IL-4-BV711 and IL-17A-BV421 [Supplementary Table 1A].

### 2.10. Immunofluorescence staining and quantification

Formalin-fixed paraffin-embedded sections were cut at 4 µm and mounted on Fisher Scientific Plus slides [Fisher Scientific]. Sections were air dried at room temperature then placed in a 60°C oven for 30 min. Deparaffinization was performed through xylene and graded alcohol baths, then slides were hydrated in de-ionized water. Antigen retrieval was performed by placing the slides in Biocare Diva solution [Biocare Medical] in the Decloaking chamber NxGen [Biocare Medical] at 125°C for a 30-s cycle. Wet slides were loaded on the Ventana Ultra Discovery [Ventana Medical Systems]. Slides were stained with anti-LAG-3 at 1:500 dilution and DAPI at 1:1000 dilution and the anti-CD4 was ready to use [RTU, Supplementary Table 1C]. Stained slides were analysed using iCyte laser scanning cytometry [LSC, ThorLabs]. The data were normalized by setting the scan area to eight regions of interest [ROIs at 500 × 574 μm] per section. ROIs were set manually to include cells in the lamina propria and epithelium and exclude gut-associated lymphoid tissue [GALT], the muscularis mucosae and submucosa. Samples used were colonic biopsies from patients with UC [*n* = 5] and normal adjacent control tissue from patients with colon cancer [*n* = 5].

### 2.11. Immunohistochemistry staining and quantification [historical cohort]

Formalin-fixed paraffin-embedded tissues were retrieved from the Oxford GI cohort and biobank, sectioned at 5 µm and collected on Superfrost glass slides [Thermofisher Scientific]. After warming to 60°C in an oven, sections were de-paraffinized in Histoclear [National Diagnostics] and rehydrated through graded ethanol baths. Antigen retrieval was performed in a Decloaking Chamber NxGen [Biocare Medical] using 10 mM citrate buffer pH 6 [Sigma-Aldrich] solution at 90°C for 20 min. Staining was then performed using the ThermoShandon Sequenza [ThermoShandon Ltd]. Non-specific FcR binding was blocked with 10% human serum [Sigma Aldrich] followed by primary antibody staining with mouse anti-LAG-3 [Lifespan Biosciences] at 1:500 dilution and anti-calprotectin [S100A, Dako] at 1:800 dilution. Slides were incubated at room temperature for 1 h or overnight for LAG-3 and 1 h for calprotectin [Supplementary Table 1D]. Respective isotype-matched control antibodies [Dako] were included. Endogenous peroxidases were blocked with 3% hydrogen peroxide [Sigma Aldrich] for 15 min. This was followed by detection using the Dako EnVision Kit [Dako] with anti-mouse/rabbit secondary antibody and visualization using the substrate-chromogen [DAB, Dako] solution for 5 min. Counterstaining was carried out in Mayer’s Haematoxylin [Sigma Life Science] for 3 s. Stained slides were mounted using Aquamount [Merck] and left to dry at room temperature.

Slides were digitally scanned using a 40× objective equivalent using the Aperio Scanscope [Leica Biosystems]. Two independent blinded observers [M.T. and L.M.W.] determined the number of cells positive for LAG-3 or calprotectin in five and three representative high-power fields [HPFs] using the scientific image-analysis program ImageJ [NIH]. Staining without association with a nucleated cell, within the crypts, the submucosa and gut-associated lymphoid tissue were excluded from analysis. Data were displayed as the average LAG-3^+^ cell count per five HPFs and the average calprotectin^+^ cell count per three HPFs.

### 2.12. Mixed lymphocyte reaction assay

PBMCs from healthy donors were isolated by Ficoll density centrifugation from peripheral blood. For the responder cells, PBMCs from individual donors were loaded with CellTrace Violet [Invitrogen]. For the stimulator cells, PBMCs from five separate donors were pooled and first depleted of T cells using anti-CD3 microbeads [Miltenyi Biotec]. CD3-depleted PBMCs at 1 × 10^6^/mL were subsequently treated with 50 µg/mL Mitomycin C [Merck Millipore] for 1.5 h at 37°C before washing twice. Responder PBMCs and pooled stimulator PBMCs were then cultured 1:1 in 96 U well plates [2 × 10^5^ per well total in quadruplicate]. An anti-LAG-3 depleting monoclonal antibody [mAb] [GSK2831781], and an antibody dependent cellular cytotoxicity [ADCC]-enhanced IgG1 control antibody [both supplied by GSK] were titrated in the assay from 10 µg/mL. Both GSK2831781 and the control antibody were ADCC-enhanced using POTELLIGENT Technology [BioWa, Inc]. After 5 days supernatants were removed and subsequently tested for IFNγ by ELISA [R&D Systems]. Cells from replicate wells were pooled and T cell proliferation and LAG-3 expression were assessed by flow cytometry. Cells were treated with green fixable Live/Dead stain [Life Technologies] and after blocking Fc receptors with Trustain [BioLegend], samples were stained with an antibody cocktail to cell surface markers CD3, CD4, CD8, CD25 [all BioLegend] and LAG-3 [Miltenyi Biotec] for 30 min at room temperature. Samples were acquired using a CANTOII flow cytometer [BD Biosciences] and data were analysed using FACS Diva software v8.0.1 [BD Biosciences]. After excluding doublets and dead cells, the live cells were gated to separate out the CD3^+^ T cells and the CD4^+^ and CD8^+^ subpopulations. The cell trace violet low [CTV^lo^, proliferated] CD4^+^ and CD8^+^ T cells were acquired into CD25/LAG-3 plots to assess depletion of the double positives.

### 2.13. Statistics

Statistical analyses were performed using GraphPad Prism version 7 [GraphPad Software]. Statistically significant *p* values are indicated as follows: not significant [ns], *p < 0.05, * *p* < 0.01, ***p < 0.001 and ****p < 0.0001. Statistical tests are specified in the figure legends. Wilcoxon’s match-pairs signed rank test was performed.

## 3. Results

### 3.1. Increased frequency of LAG-3^+^ T cells in the inflamed colon of patients with UC

**Figure 1.**
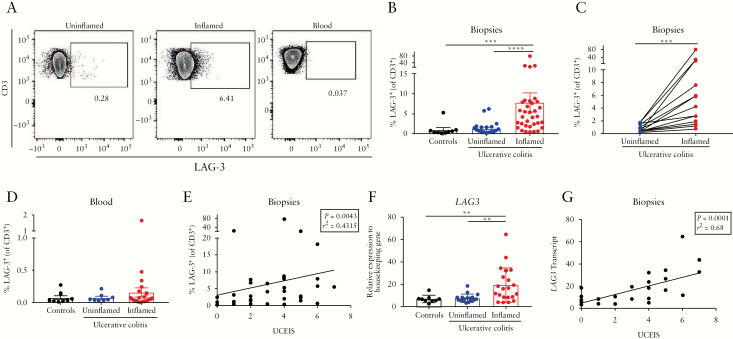
LAG-3^+^ T cells are increased in the inflamed colon of patients with UC. [A] Representative flow plots of LAG-3 staining on CD3^+^ T cells from uninflamed and inflamed colonic LPMCs, and PBMCs, from a UC patient with active disease. [B] The percentage of LAG-3^+^ cells as a proportion of CD3^+^ T cells amongst non-IBD controls [*n* = 9], UC uninflamed [*n* = 24] and UC inflamed [*n* = 34] colonic biopsies [median, IQR]. [C] Paired analysis of the percentage of LAG-3^+^ cells as a proportion of CD3^+^ T cells from uninflamed and inflamed UC biopsies [*n* = 16]. [D] The percentage of LAG-3^+^ cells as a proportion of CD3^+^ T cells from PBMCs of non-IBD controls [*n* = 8], UC uninflamed [n = 8] and UC inflamed [*n* = 24] samples [median, IQR]. [E] Correlations of the frequency of CD3^+^LAG-3^+^ LPMCs in all patients with UC [uninflamed and inflamed] vs UCEIS [*n* = 42]. [F] Transcriptional expression of colonic *LAG3* in: non-IBD controls [*n* = 9], UC uninflamed [*n* = 17] and UC inflamed [*n* = 21, nine of which are paired samples] biopsies were measured by qRT-PCR, and normalized to the housekeeping genes *POLR2G* and *POLR2J* [median, IQR]. [G] Correlation of *LAG3* transcript from all patients with UC [uninflamed and inflamed] with UCEIS. **p < 0.01, ***p < 0.001 and ****p < 0.0001. Comparisons for continuous data were performed using the Mann–Whitney U test, paired analyses with the Wilcoxon test, and correlations with the Spearman test.

We first sought to determine whether the proportions of LAG-3^+^ cells were altered in inflamed compared to uninflamed bowel, in patients with UC, and compared to uninflamed bowel from patients without UC. The characteristics of subjects are described in Table 1. Participants with UC had moderately active disease with a median UCEIS score of 4/8 and a Nancy score of 3/4, and had been treated with a range of biologics, anti-inflammatory and immunosuppressant drugs. Flow cytometry plots for LAG-3 staining on CD3^+^ T cells from the uninflamed and inflamed LPMCs and corresponding PBMCs are shown in Figure 1A and the fluorescence minus one [FMO] for LAG-3 is shown in Supplementary Figure 2A. The frequency of CD3^+^LAG-3^+^ cells was increased [median 4.21%, 95% confidence interval 2.5–6.79] in inflamed tissue compared to uninflamed tissue [0.81%, 0.4–1.51] and from patients without IBD [0.35%, 0.31–0.91, Figure 1B]. Among paired samples [uninflamed and inflamed tissue obtained from the same patient], the proportion of LAG-3^+^ T cells was increased in all 16 UC inflamed samples [Figure 1C]. Corresponding blood samples from the same individuals, however, showed no difference in CD3^+^LAG-3^+^ T cells, which constituted no more than 1.5% of circulating T cells in any individual [Figure 1D]. There was no correlation between LAG-3 expression in the intestine and the blood [*p* = 0.69, *r*^2^ = 0.12]. The percentage of CD3^+^LAG-3^+^ T cells in patients with UC correlated positively with endoscopic severity [Figure 1E]. Consistent with the flow cytometry data, expression of *LAG3* transcripts was increased in inflamed colonic biopsies of patients with UC relative to both uninflamed tissue and non-IBD control tissue [Figure 1F]. Furthermore, the transcript levels of *LAG3* correlated positively with the UCEIS [Figure 1G] and Nancy histological index [Supplementary Figure 2B]. As a result, these data suggest LAG-3 expression and frequency identify activated T cells and correlate with intestinal inflammation.

### 3.2. LAG-3^+^ cells are enriched within effector memory and central memory T cell populations

**Figure 2.**
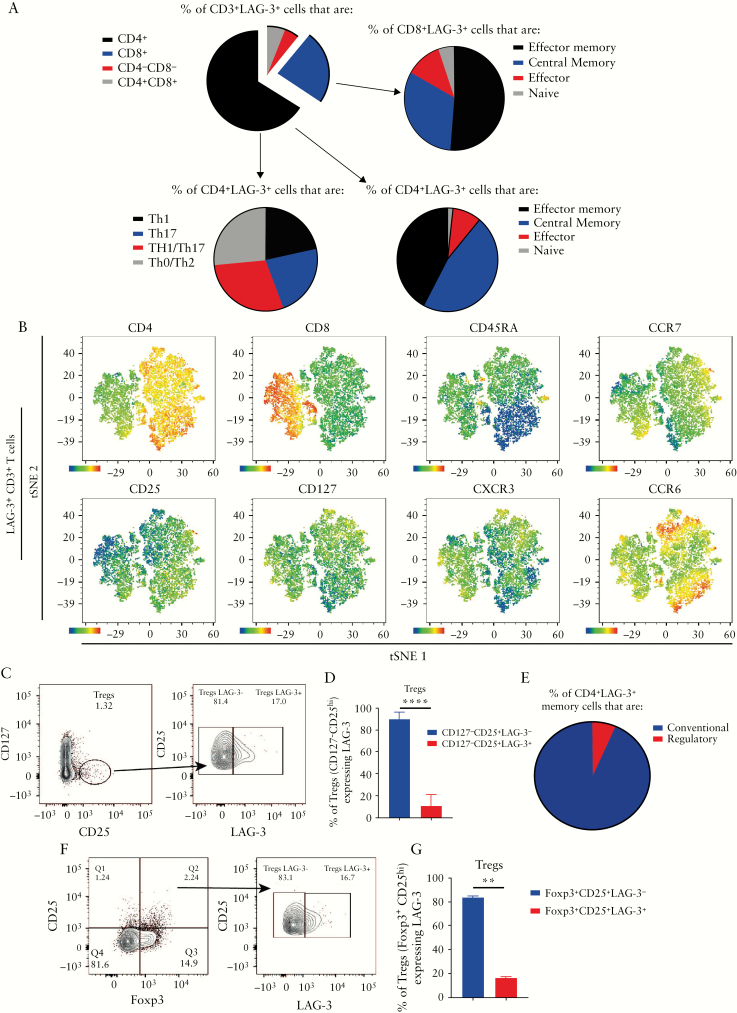
LAG-3^+^ cells are enriched within the effector memory and central memory T cell populations. [A] Pie charts illustrating the proportion of CD3^+^LAG-3^+^ T cells that belong to the subset of: CD4^+^, CD8^+^, naïve [CD45RA^+^CCR7^+^], antigen experienced/memory [CD45RA^−^CCR7^+/−^] and T helper cells. Charts were generated using the averaged percentage of each T cell subset expressing LAG-3 from the UC inflamed group [*n* = 34]. [B] t-SNE visualization of mucosal T cell clusters [flow panel 1] within the LAG-3^+^ cells of three UC patients with active disease. [C] Representative plot of LPMCs from a patient with active UC stained for surface Treg markers [CD4^+^CD127^−^CD25^hi^] and the percentage of LAG-3^+^ and LAG-3^−^ cells amongst this population. [D] Bar graph showing the proportion of lamina propria Tregs that are LAG-3^+^ and LAG-3^−^ from the UC patients with active UC [median, IQR, *n* = 34]. [E] Pie chart highlighting the proportion of CD4^+^LAG-3^+^ antigen experienced/memory cells [CD45RA^−^] within the active UC cohort [*n* = 34] that are conventional [CD127^+/−^CD25^−^] or regulatory [CD127^−^CD25^hi^]. [F] Representative plot of intranuclear Foxp3 staining of lamina propria CD4^+^ T cells from a patient with active UC and the percentage of LAG-3^+^ and LAG-3^−^ cells amongst this population. [G] Bar graph showing the proportion of lamina propria Foxp3^+^ Tregs that are LAG-3^+^ and LAG-3^−^ from patients with active UC [mean ± SEM, *n* = 3]. **p < 0.01, ****p < 0.0001. Comparisons of data were performed using paired t tests.

To characterize the subsets of T cells expressing LAG-3, detailed flow cytometry panels were designed and the schematics and full gating strategies are shown in Supplementary Figure 3A-C. Because LAG-3^+^ cells are predominantly present within inflamed tissue as shown in Figure 1B, the proportions of LAG-3 on naïve, memory and T helper subsets from inflamed UC samples [*n* = 34] were averaged and are presented in Figure 2A. LAG-3 was expressed on both CD4^+^ and CD8^+^ T cells, with the former predominating. Within both these subsets, most LAG-3^+^ cells were CD45RA^−^ and CCR7^+/−^, suggesting effector memory and central memory phenotypes.^36,37^ CD45RA^−^ cells are memory cells and therefore antigen experienced cells. A minority of LAG-3^+^ cells were CD45RA^+^, which is consistent with LAG-3 being expressed on activated cells rather than naïve subsets. LAG-3 expression was found on all subsets of T helper cells, at similar frequencies: Th1 [CXCR3^+^CCR6^−^, 1.58%], Th17 [CXCR3^−^CCR6^+^, 2.12%], Th1/Th17^38–41^ [CXCR3^+^CCR6^+^, 1.94%] and Th0/2 [CXCR3^−^CCR6^−^, 1.64%] [Figure 2A]. The breakdown of T cell populations within the inflamed biopsies is detailed in Supplementary Table 3A. Within the LAG-3^+^ cells, t-SNE plots from three patients with active UC were combined and highlight the major T cell populations expressing LAG-3 [Figure 2B].

The active UC samples [*n* = 34] were further characterized for gut homing and mucosal-associated markers. The gating strategy and breakdown of the T cell populations are detailed in Supplementary Figure 3D, E and Supplementary Table 3B. Within the LAG-3^+^ cells, t-SNE plots from three patients with active UC were combined and the T cell populations that are enriched for LAG-3 expression are shown in Supplementary Figure 3F. CD161, integrin β7, integrin αE and CCR9 are all expressed within the LAG-3^+^ T cells and these markers are important in T cell trafficking to the gut.^42–45^ As LAG-3 expression has previously been reported on Treg cells,^46–51^ we characterized these by flow cytometry. Surface staining for Tregs as CD4^+^CD45RA-CD127^−^CD25^hi^ cells in inflamed tissue [Figure 2C] showed that a median of 90% of these cells were LAG-3^−^ [Figure 2D, *n* = 34]. The main population of LAG-3^+^ cells comprised of conventional T cells [CD45RA^−^CD127^+/−^CD25^−^], and Treg cells [CD45RA^−^CD127^−^CD25^hi^] were a small minority in the CD4 memory compartment [Figure 2E]. As cell surface markers tend to overestimate the number of Treg cells, we sought to confirm this result using intranuclear staining for Foxp3 [Figure 2F], which validated the finding that only a small population of Tregs in the inflamed bowel were LAG-3^+^ [Figure 2G, *n* = 3]. Altogether, within the inflamed UC tissue, LAG-3 is expressed predominately on the conventional effector memory and central memory CD4^+^ and CD8^+^ T cells.

### 3.3. *LAG3*^+^ cells are a heterogeneous population of cytokine-expressing activated T cells

To further characterize LAG-3^+^ T cells within the inflamed intestine, we performed single-cell RNA-sequencing of CD3^+^ T cells from colonic LPMCs of four patients with active UC [Supplementary Table 2]. Unsupervised clustering analysis of the four combined samples [11 708 cells total] identified 15 transcriptionally distinct clusters of colonic T cells [Supplementary Figure 4A]. Comparison of the transcriptional state of colonic T cells from ascending [*n* = 1] and distal [*n* = 3] colon samples revealed major differences between T cells from these two anatomical sites [Supplementary Figure 4B, C]. T cells from the ascending colon were essentially unique to clusters 4 and 14, and conversely were absent from clusters 0, 1, 5, 6, 9 and 13. Given this distinction between the ascending and distal colon samples, and that we had data on the ascending colon from only one patient, we decided to focus on the three distal colon samples for further analysis.

**Figure 3.**
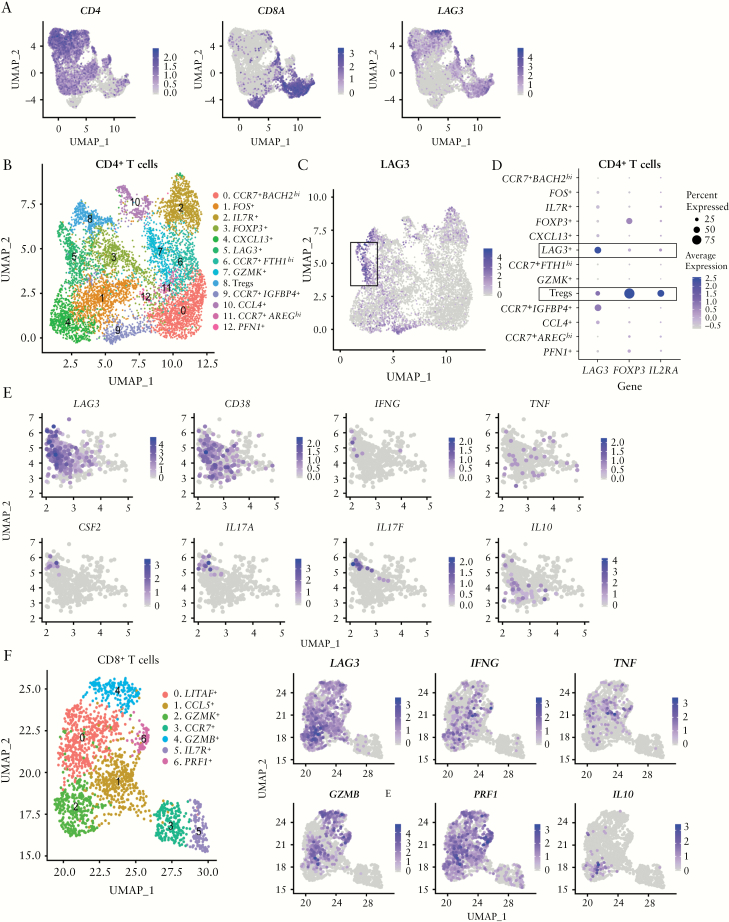
*LAG3* ^+^ cells are a heterogeneous population of cytokine-expressing activated T cells. Single-cell RNA-sequencing of CD3^+^ LPMCs from UC patients with active distal disease [*n* = 3]. [A] UMAP [uniform manifold approximation and projection for dimension reduction] of colonic CD3^+^ T cells showing the expression of *CD4*, *CD8A* and *LAG3*. [B] Clusters of colonic CD4^+^ T cells as visualized by UMAP. [C] Expression of *LAG3* in the CD4^+^ T cell clusters. [D] Dot plot showing the expression of *LAG3* and the regulatory T cell markers *FOXP3* and *IL2RA* in the CD4^+^ T cell clusters. [E] Expression of *LAG3*, T helper cell cytokines and the activation marker *CD38*, by cluster 5 CD4^+^ T cells. [F] Clusters of colonic CD8^+^ T cells as visualized by UMAP. [G] Expression of *LAG3*, *IFNG*, *TNF*, *GZMB*, *PRF1* and *IL10* in the CD8^+^ T cell clusters.

Within the distal colon samples, *LAG3* was expressed within both CD4^+^ and CD8^+^ T cells **[**Figure 3A**]**. To characterize these *LAG3*-expressing T cells further, CD4^+^ and CD8^+^ T cells were analysed separately. CD4^+^ T cells comprised of 13 clusters **[**Figure 3B**]**. *LAG3* was most highly expressed in cluster 5 and showed low expression in Treg cells [cluster 8; Figure 3C, D]. CD4^+^ T cells within cluster 5 expressed an array of cytokines [*IFNG*, *TNF*, *CSF2*, *IL17A, IL17F* and *IL10*] associated with different T helper cell populations, including Th1 and Th17 **[**Figure 3E**]**. The majority of these cytokine genes were expressed by separate cells within the cluster, suggesting a heterogeneous CD4^+^ T cell group with an overall activated phenotype, as supported by their unanimous expression of the activation marker *CD38* [Figure 3E]. Within the seven clusters of CD8^+^ T cells [Figure 3F], the clusters with the highest *LAG3* expression [clusters 0, 1, 2, 4 and 6] exhibited an activated cytotoxic phenotype, with expression of *IFNG*, *TNF*, *PRF1* and *GZMB*, in contrast to the two *LAG3*^−^ clusters [clusters 3 and 5, Figure 3G]. Pathway enrichment analysis of the genes upregulated within the CD4^+^ and CD8^+^ T cell clusters with the highest *LAG3* expression, namely CD4^+^ cluster 5 and CD8^+^ cluster 2, identified enriched expression of TCR and cytokine signalling pathways [Supplementary Figure 5A, B]. Overall, the single-cell RNA-sequencing data demonstrate that *LAG3* expression is enriched within activated, cytokine-expressing, T cells.

### 3.4. LAG-3^+^ colonic T cells predominantly secrete IFNγ and IL-17A

**Figure 4.**
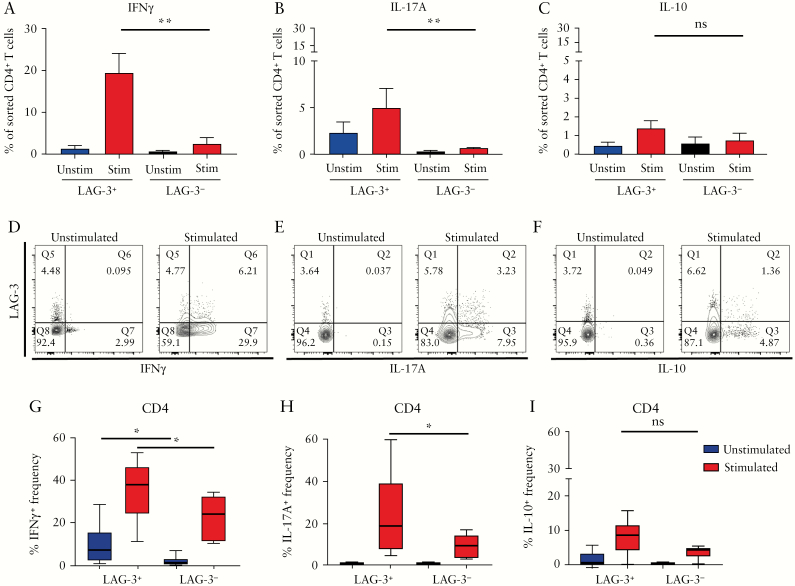
LAG-3^+^ colonic T cells predominantly secrete IFNγ and IL-17A. *Ex vivo* stimulated PBMCs from healthy controls [*n* = 5] were sorted into LAG-3^+^ and LAG-3^−^ subsets before re-stimulation and IC staining for [A] IFNγ, [B] IL-17A and [C] IL-10. LPMCs from patients with active UC were extracted from colonic biopsies and stained for LAG-3, IFNγ, IL-17A and IL-10. [D–F] Representative FACS plots and corresponding graphs [G–I] for PMA and ionomycin-stimulated [red bars, right] and unstimulated [blue bars, left] conditions. *p < 0.05 and **p < 0.01. Comparisons for continuous data were performed using the Mann-Whitney U test, and paired analyses with the Wilcoxon test.

To validate the single-cell RNA-sequencing data, we first investigated the cytokine profile of LAG-3^+^ cells in the blood. LAG-3^+^ and LAG-3^−^ T cells were sorted from *in vitro* stimulated PBMCs from healthy controls, using anti-CD3 and anti-CD28 [*n* = 5]. There was a substantial increase in the frequency of IFNγ ^+^ and IL-17A^+^ cells in the CD4^+^LAG-3^+^ compartment compared to the CD4^+^LAG-3^−^ compartment, a moderate increase in IL-10 [Figure 4A–C**and**Supplementary Figure 6A], and low or undetectable frequencies of IL-4, IL-22 and GM-CSF-positive cells in general [data not shown]. To examine cytokine production by LAG-3^+^ cells from inflamed colonic tissue, LPMCs were isolated from colonic biopsies of patients with active UC [*n* = 6]. Cytokine secretion assays were used to determine production of IFNγ, IL-17A and IL-10 in control and PMA/ionomycin-stimulated LAG-3^+^ and LAG-3^−^ T cells. Without stimulation, the proportion of CD4^+^LAG-3^+^ cells producing IFNγ was higher than for LAG-3^−^ cells. Stimulation induced production of all three cytokines from all T cells; however, proportionally, LAG-3^+^ cells showed greater frequencies of IFNγ and IL-17A compared to LAG-3^−^ cells, while IL-10 production was not statistically significant between LAG-3^−^ and LAG-3^+^ cells [Figure 4G–I and Supplementary Figure 6B–D]. Representative flow plots from an inflamed UC sample are shown in Figure 4D–F. Thus, stimulated LAG-3^+^ colonic T cells present a Th1/Th17 phenotype based on their cytokine profile.

### 3.5. LAG-3 cell numbers correlate with endoscopic inflammation and are reduced in responders to biological therapy

**Figure 5.**
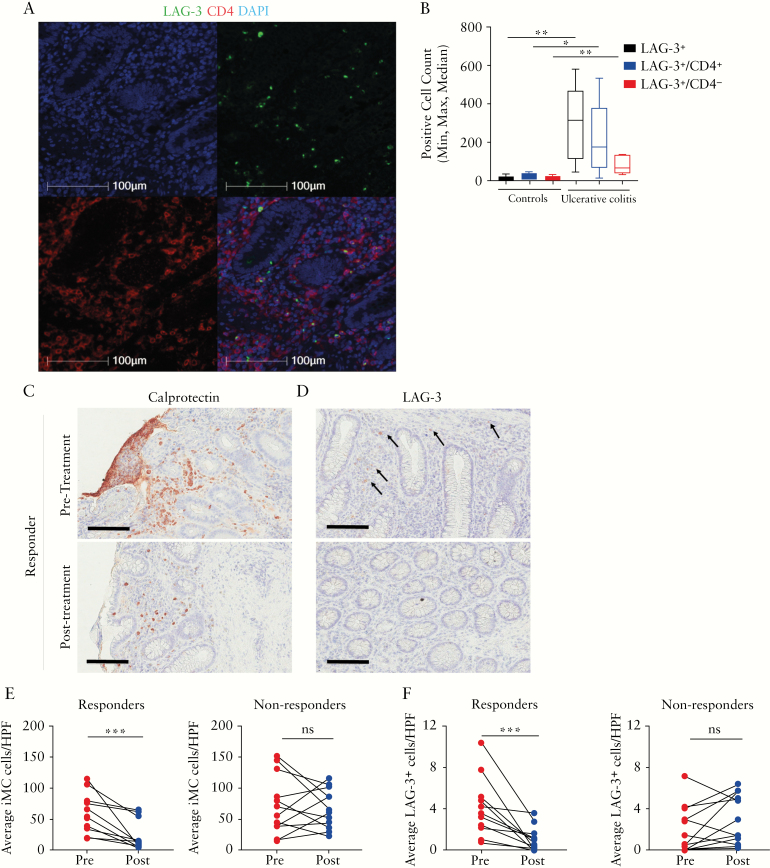
LAG-3 cell numbers correlate with endoscopic inflammation and are reduced in responders to biological therapy. [A] Representative laser scanning cytometry images of nuclei [blue, top left], LAG-3 [green, top right], CD4 [red, bottom left] and dual stain [yellow, bottom right] in UC colonic mucosa. All images are shown at 100× magnification. [B] Box plot for LAG-3 and CD4-positive cell counts in UC and control colonic mucosa [*n* = 5]. Representative immunohistochemistry [IHC] images of colonic mucosa stained for [C] intra-mucosal calprotectin [iMC] and [D] LAG-3 from a patient with active UC who responded to biological therapy, before and after treatment. Black arrows point to positive staining. [E] Average iMC expression per three high power fields [HPFs] and [F] average LAG-3^+^ cell numbers per five HPFs were enumerated in responders [*n* = 11] and non-responders [n = 12]. Not significant [ns] and ***p < 0.001. Data comparisons were performed using the Mann–Whitney U test [B] and the Wilcoxon paired test [E and F].

Representative images of CD4 and LAG-3 immunofluorescence staining in colonic tissue of patients with UC are shown in Figure 5A. CD4^+^LAG-3^+^ cells were localized in the lamina propria with increased CD4^+^ cells present in the UC sample compared to controls. The median number of LAG-3^+^ cells in the mucosa was increased in the UC samples (315 cells, interquartile range [IQR] 48–582, *n* = 5) compared to control tissue [18 cells, IQR 4–44, n = 5, Figure 5B]. To determine the relationship of colonic LAG-3^+^ cell numbers with other markers of disease activity, and the impact of UC treatment, we retrieved biobanked UC colonic biopsy samples and quantified LAG-3^+^ cells by immunohistochemistry. Baseline clinical and demographic data for the historical cohort are presented in Table 2. Patients were stratified by their response to biological therapy [defined by reduction in UCEIS and Nancy score, see the Methods section]. LAG-3^+^ cell numbers were reduced in patients who responded to biological treatment [anti-TNF or vedolizumab], but not in non-responders [Figure 5D, F]. Similar findings were also observed for mucosal calprotectin, a granulocyte marker^52^ [Figure 5C, E]. Isotype control staining is shown in Supplementary Figure 7A. Overall, LAG-3 expression is present during colonic inflammation and interestingly is reduced or absent when mucosal healing is achieved in UC.

### 3.6. Anti-LAG-3 depletes LAG-3^+^ T cells, inhibits proliferation and IFNγ production in an MLR

**Figure 6.**
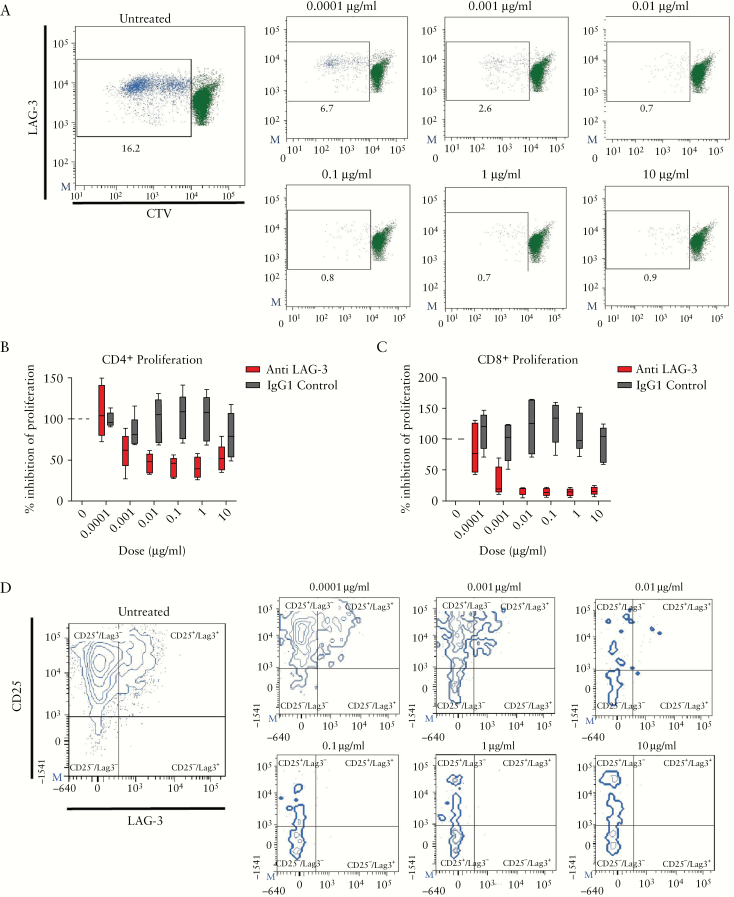

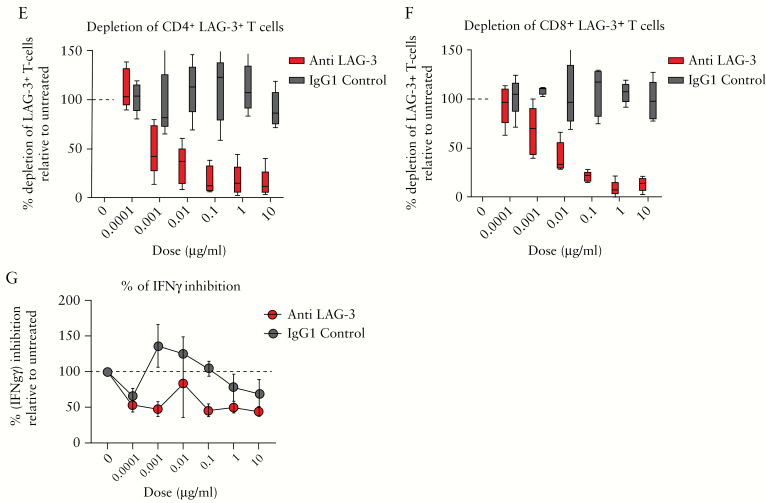
Anti-LAG-3 depletes LAG-3^+^ T cells, inhibits proliferation and IFNγ production in a mixed lymphocyte reaction [MLR]. [A] Representative flow plots depicting proliferating Cell Trace Violet low CD8^+^ T cells in an MLR. Percentage of proliferating CD4^+^ [B] and CD8^+^ [C] T cells in GSK2831781-treated samples relative to untreated control in an MLR. [D] Representative flow plots of activated CD25^+^LAG-3^+^ T cells in an MLR. Percentage of CD4^+^LAG-3^+^ [E] and CD8^+^LAG-3^+^ [F] T cells in GSK2831781-treated samples relative to untreated baseline in an MLR. [G] Percentage of IFNγ inhibition in GSK2831781-treated samples relative to untreated control in an MLR. Red boxes represent anti-LAG-3 [GSK2831781]-treated samples and grey boxes denote IgG1 controls. Results shown are representative of two independent experiments with *n* = 4–5 donors respectively.

To investigate the role of LAG-3, a mixed lymphocyte reaction [MLR] with and without the addition of a depleting LAG-3 antibody [GSK2831781] was performed. Healthy PBMCs were cultured for 5 days with a depleting anti-LAG-3 mAb. The frequency of proliferating responder cells labelled with CTV^lo^ reduced with increasing concentrations of the drug [red bars] compared to the untreated baseline and the IgG1 control [grey bars, Figure 6A, B]. This reduction in proliferation was evident in both the CD4^+^ [Figure 6A, B] and CD8^+^ [Figure 6C] responder T cells. Keeping with the mechanism of action of the drug, GSK2831781 treatment led to a depletion of LAG-3^+^CD4^+^ [Figure 6D, E] and CD8^+^ T cells [Figure 6F], in a dose-dependent manner. To test if this reduction in the frequency of proliferating T cells resulted in changes in pro-inflammatory cytokines, we measured IFNγ levels in the supernatants from the MLRs. Depletion with GSK2831781 manifested in an ~50% inhibition of IFNγ relative to baseline untreated and non-specific IgG1 controls [Figure 6G]. In conclusion, depleting LAG-3^+^ cells in an *in vitro* setting eliminates the activated proliferating T cells.

## 4. Discussion

LAG-3 was originally identified as a marker of T cell activation, and expression is rapidly induced on peripheral blood T cells and NK cells by TCR stimulation or cytokine stimulation.^53–55^ Consistent with these initial findings, our results demonstrate an increased frequency of effector memory and central memory LAG-3^+^ T cells in inflamed colonic mucosa from patients with UC, relative to endoscopically uninflamed regions and to biopsies from control subjects. Within the LAG-3^+^ subset, there was enrichment of cells producing IFNγ and IL-17A. Three lines of evidence reinforced these results: flow cytometry, single-cell RNA-sequencing and qRT-PCR. We also found that this increased frequency was restricted to the gut, because peripheral blood T cells were indistinguishable from subjects without colonic or other inflammation. LAG-3^+^ cells also expressed CD161, CCR9, integrin αE and integrin β7, suggesting that LAG-3 expression may be induced locally within the inflamed milieu of the lamina propria. The expression of *LAG3* within the activated cytokine-expressing T cell clusters and the correlation with endoscopic and histological disease inflammatory activity, as well as response to treatment, further supports the concept that LAG-3 is a marker of activated tissue cells in this context.

LAG-3 has previously been described on different T cell subsets,^54–56^ including Treg cells [Foxp3^+^CD25^hi^CD127^lo^],^46,51,57^ Tr1 cells [Foxp3^−^CD25^lo^IL-10^+^],^48,49,58,59^ and exhausted tumour infiltrating lymphocytes [CD8^+^PD-1^+^],^60^ as well as NK cells^53^ and B cells.^61^ These studies have largely been conducted in murine models or using healthy human blood or tissue. Natural Tregs [CD4^+^CD25^hi^, 5–10% of blood] do not express LAG-3, as depleting these cells with a cytotoxic antibody would be detrimental for IMID. Only a minority of antigen-experienced induced Tregs in human tumours^62,63^ or, in this research, active UC tissue express LAG-3.

We further considered the possibility that LAG-3 might be expressed on Tr1 cells in the inflamed mucosa. These cells do not typically express IL-17A [which is produced by ~20% of LAG-3^+^ CD4^+^ T cells], and the mean proportion of IL-10-secreting LAG-3^+^ cells was ~10%, consistent with other studies.^59^ This suggests that, at sites of active inflammation, LAG-3^+^ cells have predominantly effector rather than suppressor phenotypes. This is consistent with the observations that antibodies that deplete LAG-3^+^ T cells prolong transplant survival in a cardiac allograft model^21^ and produce sustained abrogation of T cell-driven inflammation in a non-human primate delayed-type hypersensitivity challenge.^22^ The fact that this mAb ameliorates rather than exacerbates pathologies in preclinical models has also recently been translated into humans where a beneficial effect in plaque psoriasis was observed.^64^

Our results are also consistent with a recent publication that showed while LAG-3 marks Tr1 cells in mice in conjunction with CD49b, surface LAG-3 only marked 1.6% of human Tr1 cells. Intriguingly the study also showed that human Tr1 cells overexpressed LAG-3 transcript, which might explain why this marker was originally identified as a putative Tr1 marker.^48,65^ A recent study exploring the heterogeneity of IL-10-positive T cells in mice and humans found that LAG-3 in combination with other inhibitors receptors was enriched on IL-10-positive T cells that are highly suppressive, especially in mice.^48^ The authors also found an intriguing reduction in the frequency of CD49b^+^ LAG-3^+^ IL-10^pos^ T cells in UC and Crohn’s disease patients relative to healthy controls yet not a general reduction in CD4^+^ IL-10^pos^ T cells in the same patients. To conclude, LAG-3 may mark a small subset of IL-10-positive T cells but in the context of a depleting mAb, we expect a dominant role for pathogenic IL-10^lo^ LAG-3^+^ T cells in IBD.

Treatment options for UC have improved over the past decade, with the introduction of novel drug mechanisms [such as anti-integrin antibodies and JAK inhibitors], as well as better understanding of how to optimize thiopurine and anti-TNF therapy.^66,67^ Nonetheless, these therapies still do not lead to high levels of mucosal healing or long-term remission amongst patients with moderate to severe disease. This leads to considerable morbidity and up to 30% lifetime requirements for colectomy,^68^ illustrating the magnitude of unmet therapeutic need.

Our therapeutic approach focused on the root cause of IMID by targeting the locally activated memory T cells expressing LAG-3. We produced GSK2831781, a cytotoxic LAG-3-specific antibody to deplete T cells where TCR signalling is ongoing, leading to LAG-3 expression as a marker of T cell activation. The root cause of most IMID is the few T cells reacting strongly to a self-antigen. Then, through epigenetic differentiation, these T cells become Th1 [dominant in multiple sclerosis],^69^ Th2 [asthma]^70^ or Th17 [systemic lupus erythematosus].^71^

Many successful treatments for ulcerative colitis, including thiopurines, calcineurin inhibitors and vedolizumab, target T lymphocytes, although none specifically blocks or eliminates activated T cells directly. Further evidence comes from an abundance of studies in animal models of experimental colitis demonstrating that T cells are sufficient to drive intestinal inflammation.^3^ The elimination of pathogenic T cell clones could have safety advantages such as avoiding widespread immunosuppression while aborting nascent immune activation, leading to rapid therapeutic responses. In a detailed analysis of LAG-3 expression in patients with UC who had received established biological therapies, expression of LAG-3 subsided in those who responded to treatment while remaining high in patients in whom the disease remained active. This promotes depletion or blockade of LAG-3^+^ cells as a viable drug target in active inflammation, including in patients who have not improved despite currently available treatments. As an *in vitro* proof of concept, we depleted LAG-3^+^ T cells using GSK2831781 in an MLR. Our results showed a reduction in both proliferation and IFNγ production, which demonstrates that pathogenic T cells are eliminated in such settings.

In summary, we have demonstrated that LAG-3 is expressed on activated T cells involved in the inflammation contributing to UC. This study allowed us to evaluate the expression of LAG-3 in blood and tissue, and to compare adjacent inflamed and uninflamed tissue from the same patient simultaneously. Furthermore, using recently developed and validated scores we correlated LAG-3 expression with endoscopic and histological markers of inflammation. Taken together, these data show that LAG-3 is locally regulated, and correlates closely with inflammation, and although it is at present unknown if this is causative, this provides a reasonable basis to explore the potential therapeutic benefit of depleting LAG-3^+^ cells. A phase II clinical trial of GSK2831781 in subjects with moderate to severe active UC [NCT03893565] has started. It is unknown if mucosal expression of LAG-3 is specific to UC and therefore further studies are planned to explore the expression of LAG-3 in ileal and colonic Crohn’s disease, and potentially in other conditions characterized by localized, immune-mediated inflammation.

## Supplementary Material

jjaa054_suppl_Supplementary_Figure_1

jjaa054_suppl_Supplementary_Figure_2

jjaa054_suppl_Supplementary_Figure_3_A_B

jjaa054_suppl_Supplementary_Figure_3_C

jjaa054_suppl_Supplementary_Figure_3_D_E

jjaa054_suppl_Supplementary_Figure_3_F

jjaa054_suppl_Supplementary_Figure_4

jjaa054_suppl_Supplementary_Figure_5

jjaa054_suppl_Supplementary_Figure_6

jjaa054_suppl_Supplementary_Figure_7

jjaa054_suppl_Supplementary_Table_1

jjaa054_suppl_Supplementary_Table_2

jjaa054_suppl_Supplementary_Table_3_A

jjaa054_suppl_Supplementary_Table_3_B

jjaa054_suppl_Supplementary_Figure_Legends

## Abbreviations

LAG-3: lymphocyte activation-3
UC: ulcerative colitis
MLR: mixed lymphocyte reaction
NK: natural killer
PD-1: programmed cell death protein-1
CTLA-4: cytotoxic T lymphocyte antigen-4
IMID: immune mediated inflammatory diseases
GI: gastrointestinal diseases
UCEIS: ulcerative colitis endoscopic index of severity
NHS: national health services
PBMCs: peripheral blood mononuclear cells
RBC: red blood cells
PBS: phosphate buffered saline
BSA: bovine serum albumin
LPMCs: lamina propria mononuclear cells
SOPR: special order research product
RIN: RNA integrity number
PMA: phorbol myristate acetate
UMI: unique molecular identifier
PCA: principal component analysis
UMAP: uniform manifold approximation and projection for dimension reduction
DAPI: 4’6-diamidino-2-phenylindole
MDM: modified dulbecco’s medium
RTU: ready to use
LSC: laser scanning cytometry
ROI: region of interest
GALT: gut associated lymphoid tissue
HPFs: high power fields
CRP: c reactive protein
iMC: intra-mucosal calprotectin
