# Supplementary figures and images for: Lymphocyte Activation Gene (LAG)-3 Is Associated With Mucosal Inflammation and Disease Activity in Ulcerative Colitis
Source: J Crohns Colitis. 2020 Mar 16;14(10):1446–61. doi: 10.1093/ecco-jcc/jjaa054 (PMC7533903; doi:10.1093/ecco-jcc/jjaa054)

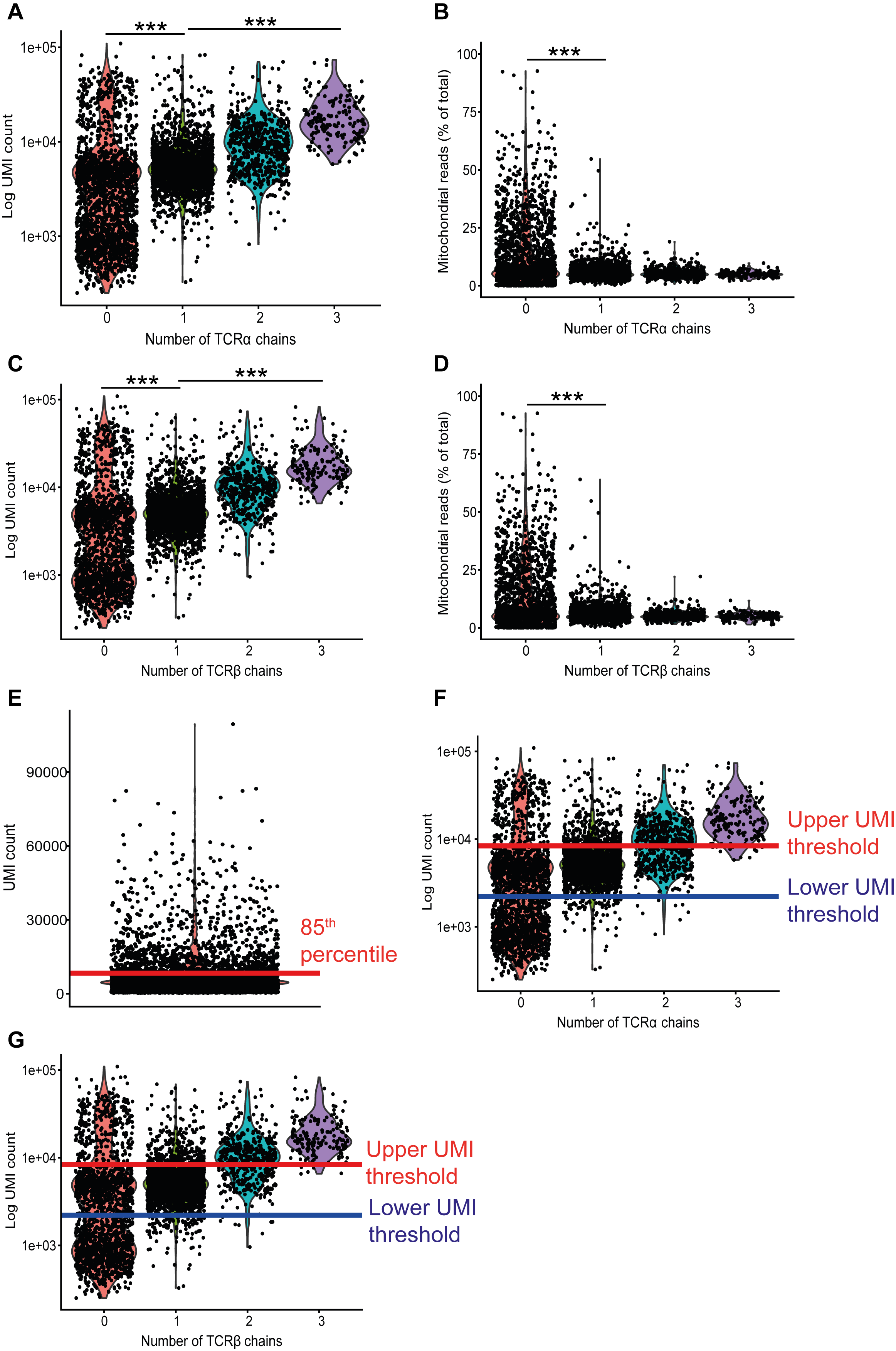

Supplement: jjaa054_suppl_Supplementary_Figure_1 [file jjaa054_suppl_supplementary_figure_1.png]

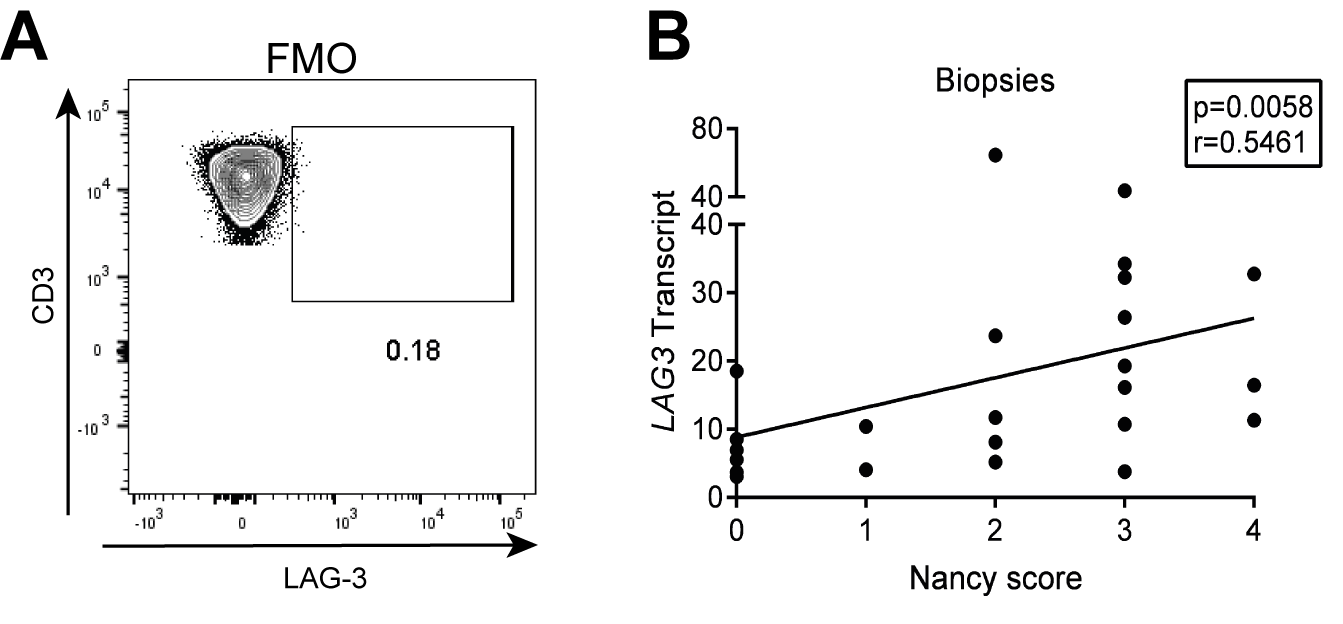

Supplement: jjaa054_suppl_Supplementary_Figure_2 [file jjaa054_suppl_supplementary_figure_2.png]

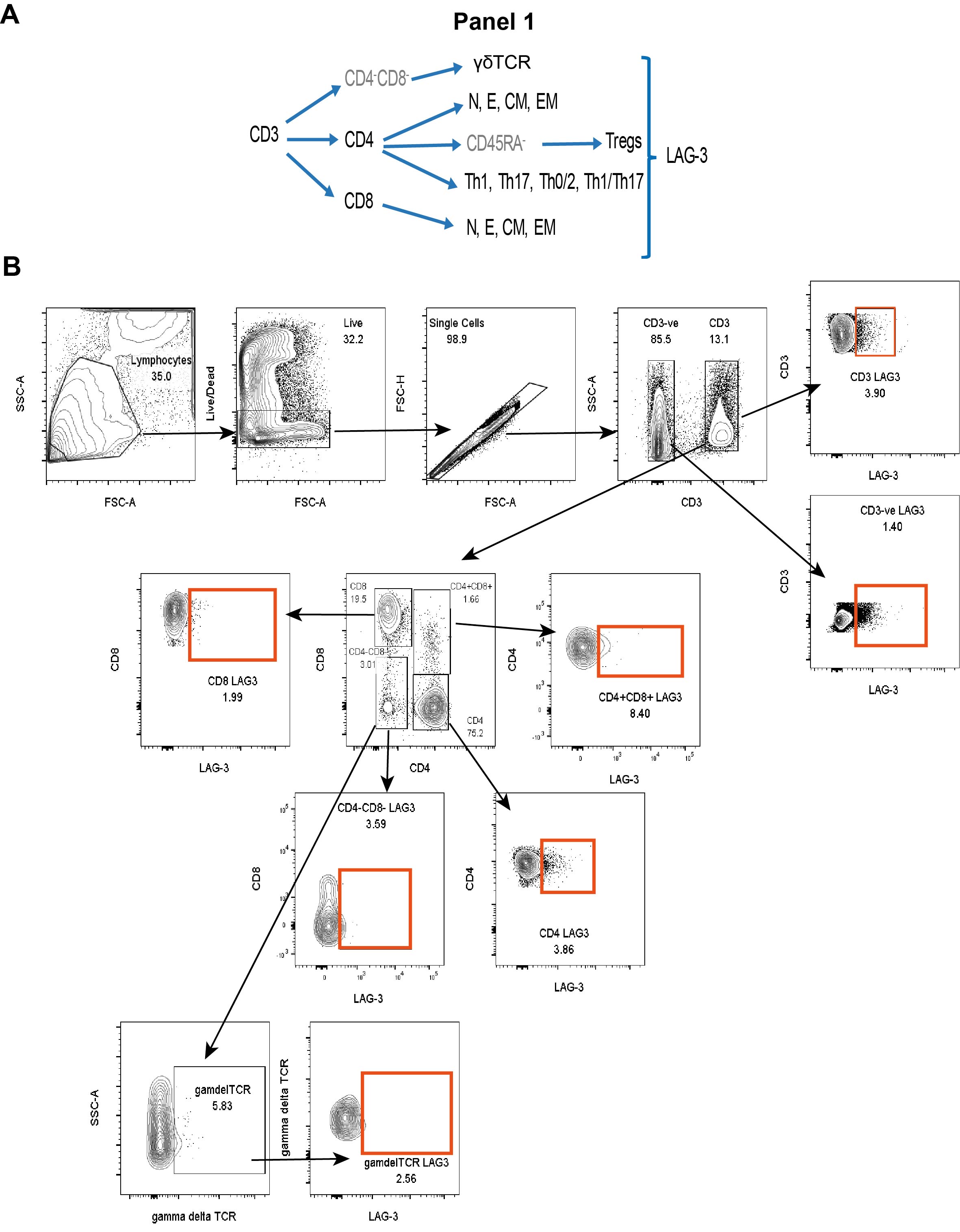

Supplement: jjaa054_suppl_Supplementary_Figure_3_A_B [file jjaa054_suppl_supplementary_figure_3_a_b.jpeg]

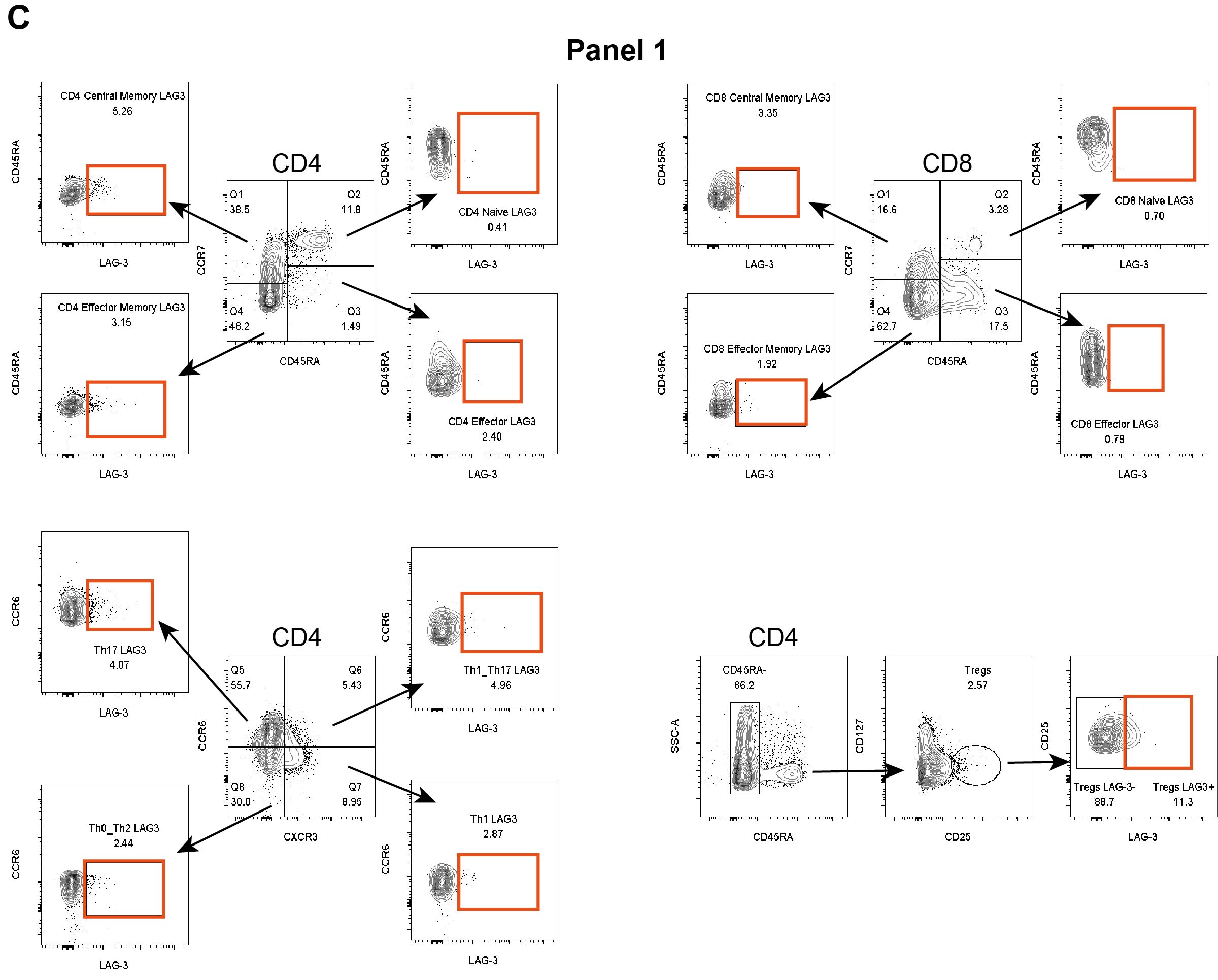

Supplement: jjaa054_suppl_Supplementary_Figure_3_C [file jjaa054_suppl_supplementary_figure_3_c.jpeg]

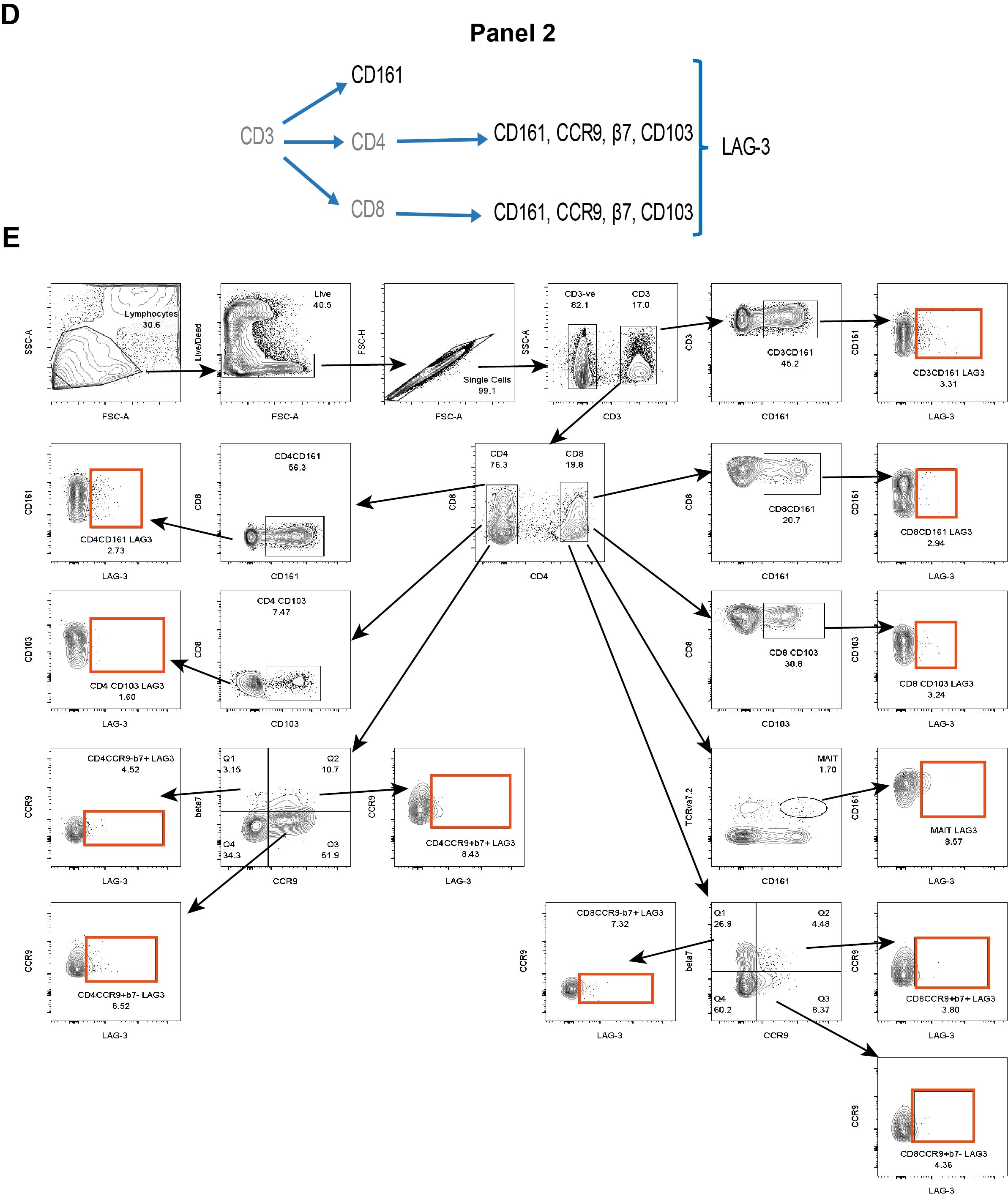

Supplement: jjaa054_suppl_Supplementary_Figure_3_D_E [file jjaa054_suppl_supplementary_figure_3_d_e.jpeg]

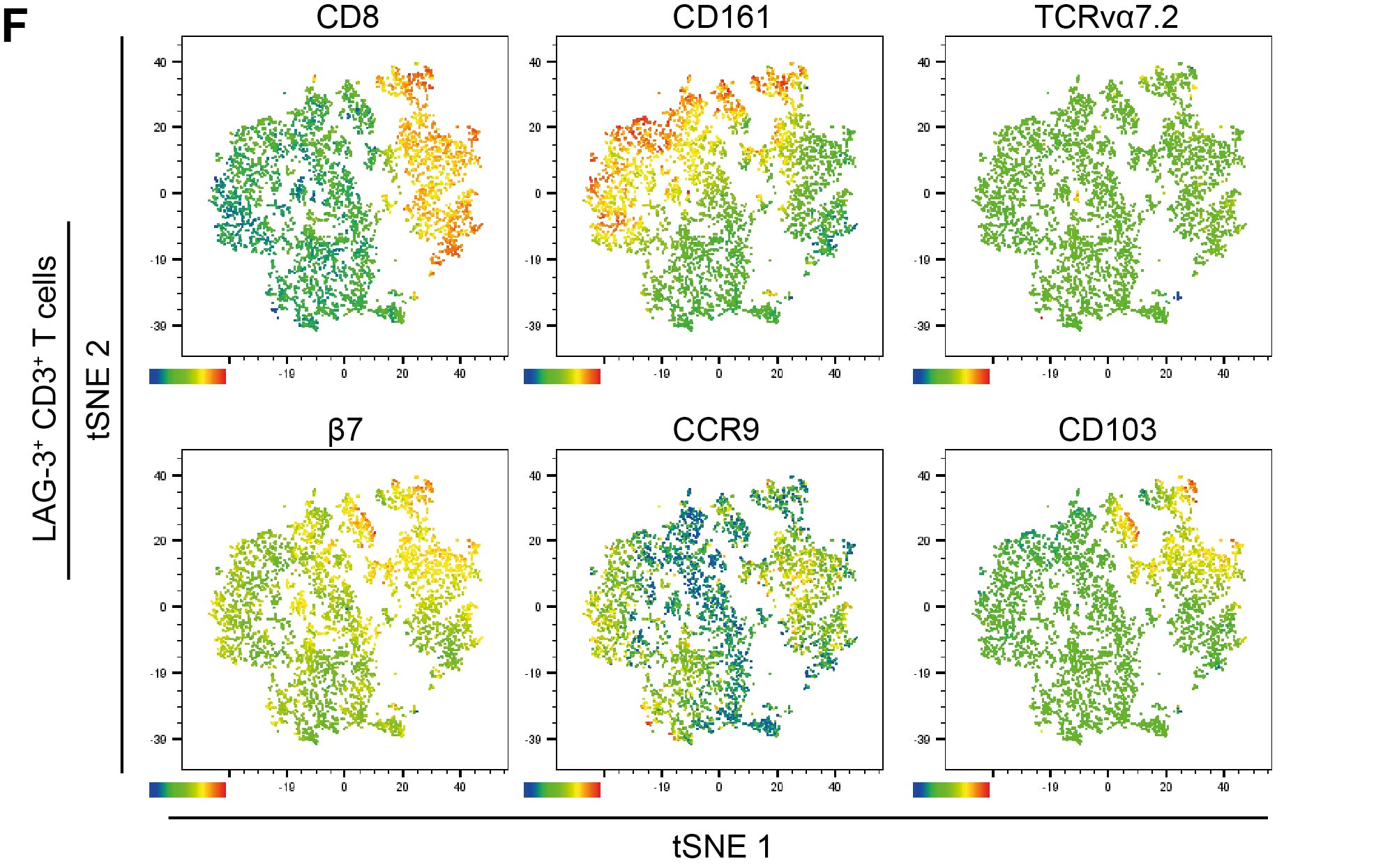

Supplement: jjaa054_suppl_Supplementary_Figure_3_F [file jjaa054_suppl_supplementary_figure_3_f.jpeg]

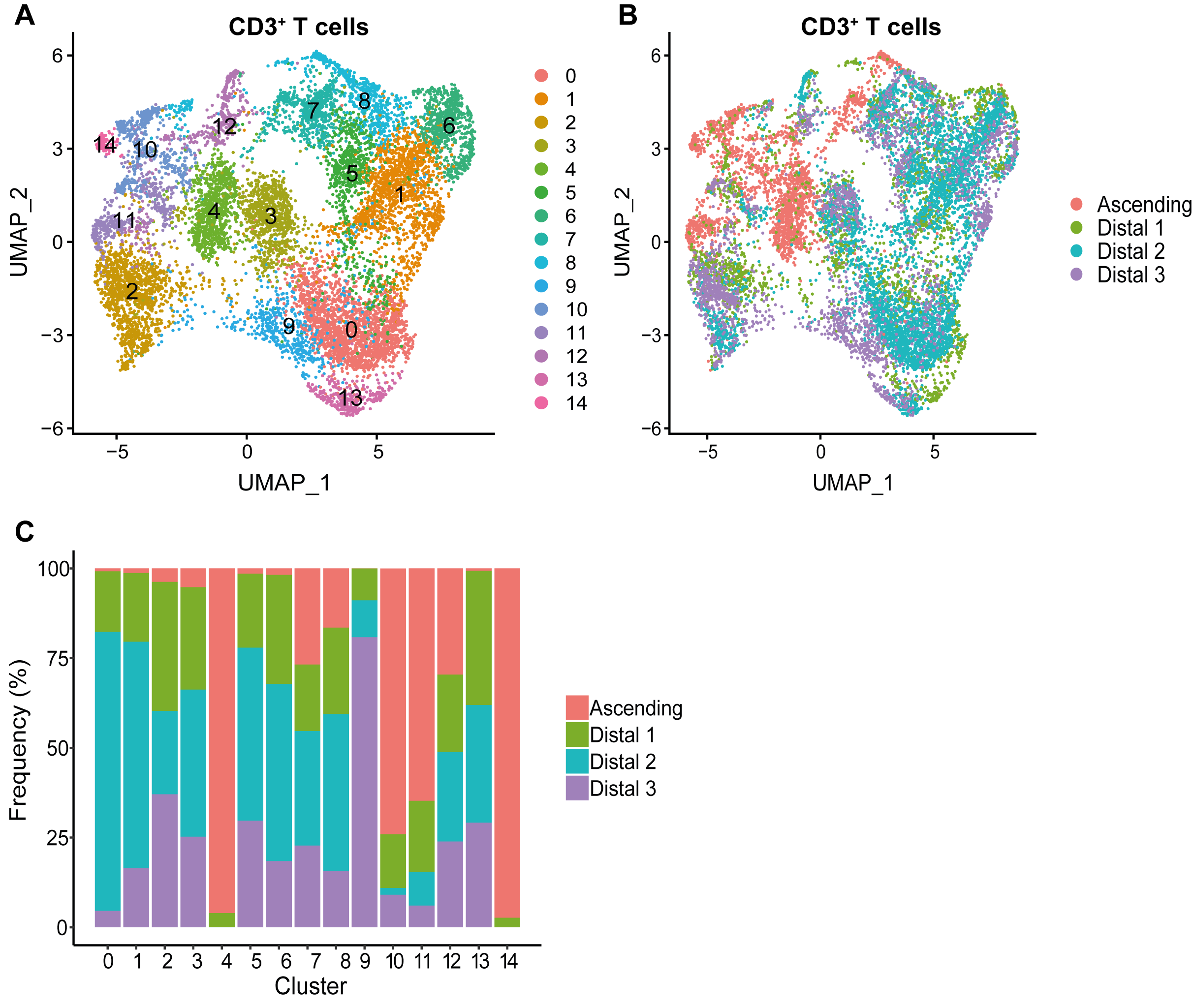

Supplement: jjaa054_suppl_Supplementary_Figure_4 [file jjaa054_suppl_supplementary_figure_4.png]

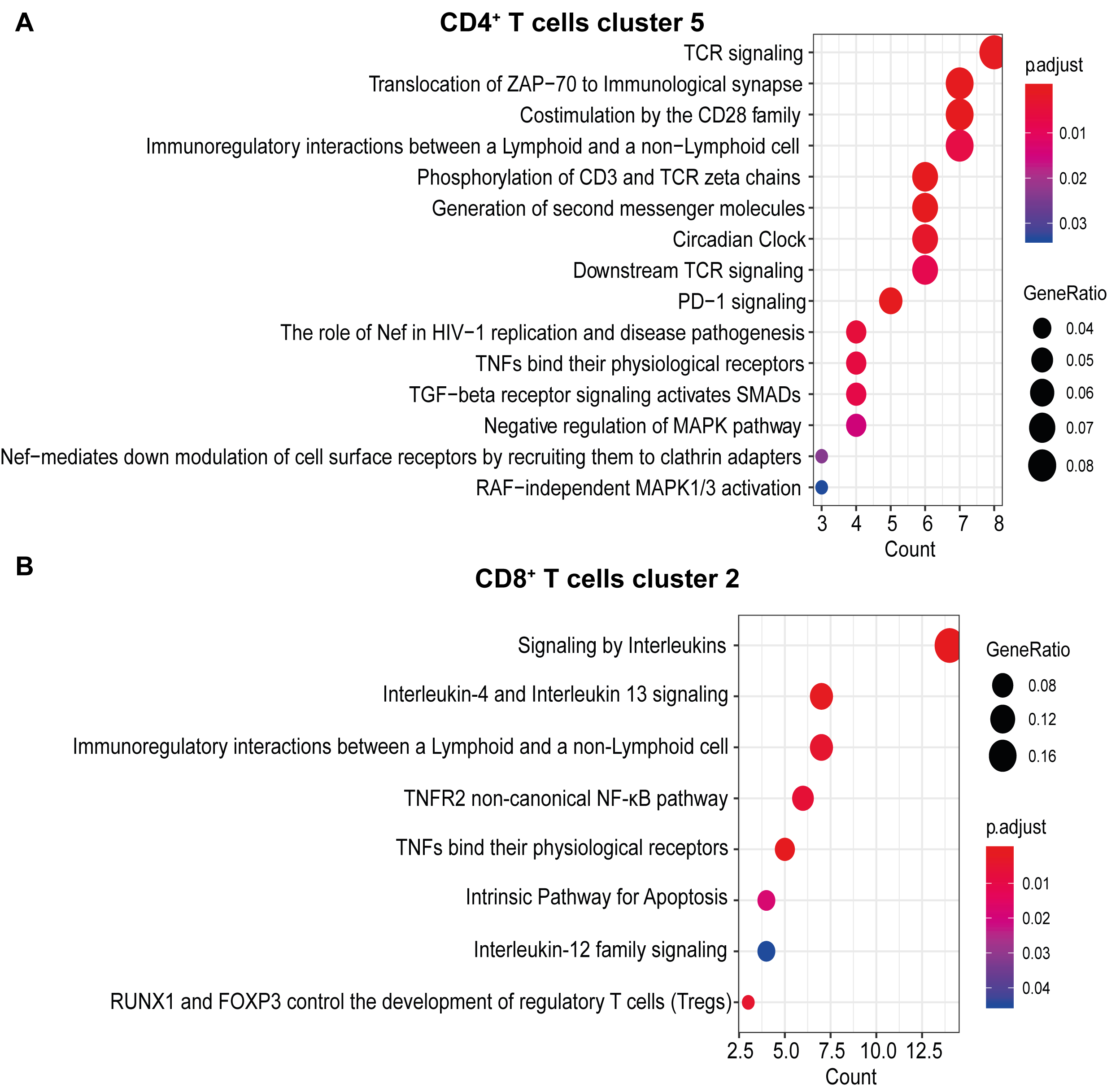

Supplement: jjaa054_suppl_Supplementary_Figure_5 [file jjaa054_suppl_supplementary_figure_5.png]

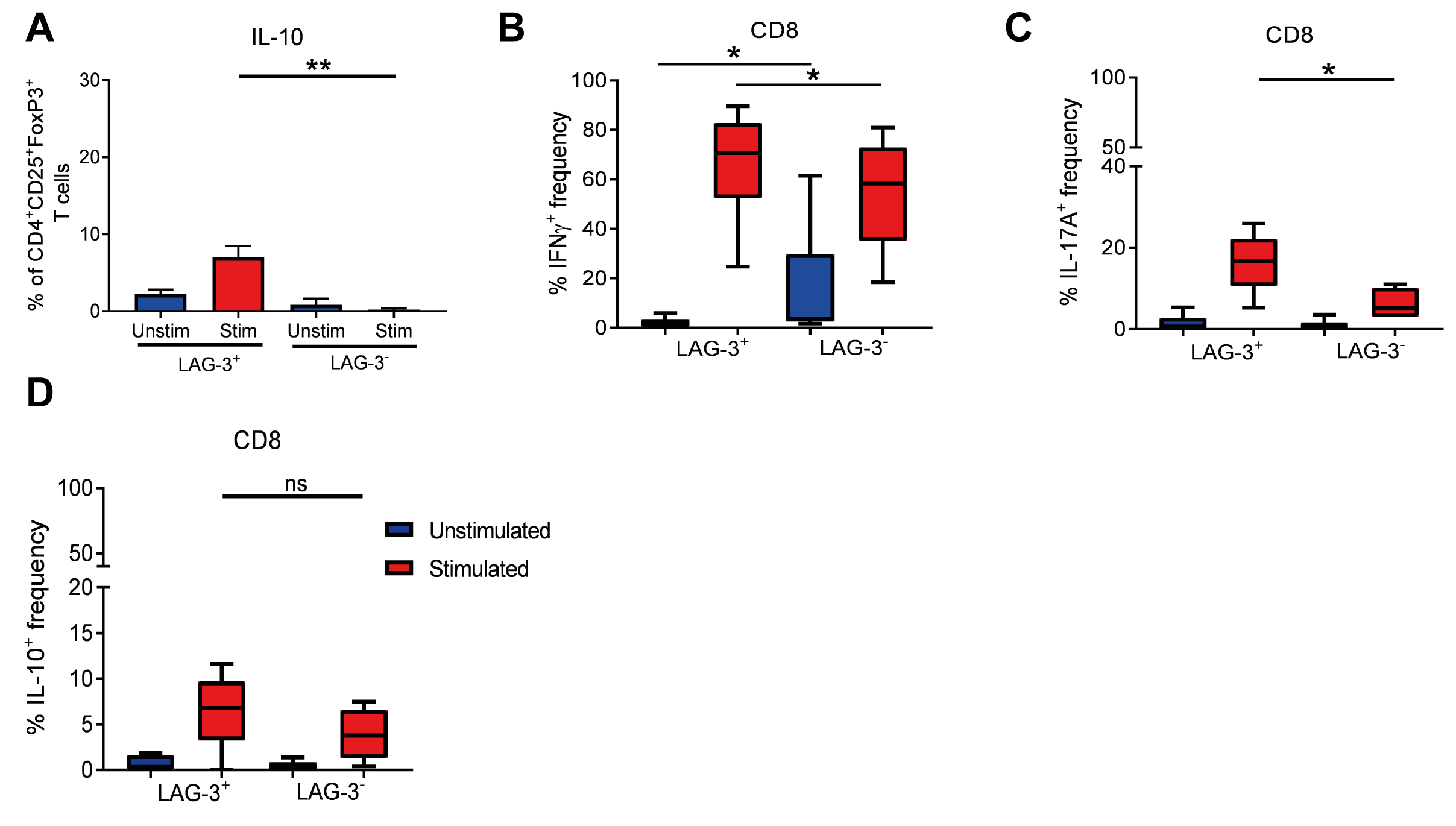

Supplement: jjaa054_suppl_Supplementary_Figure_6 [file jjaa054_suppl_supplementary_figure_6.png]

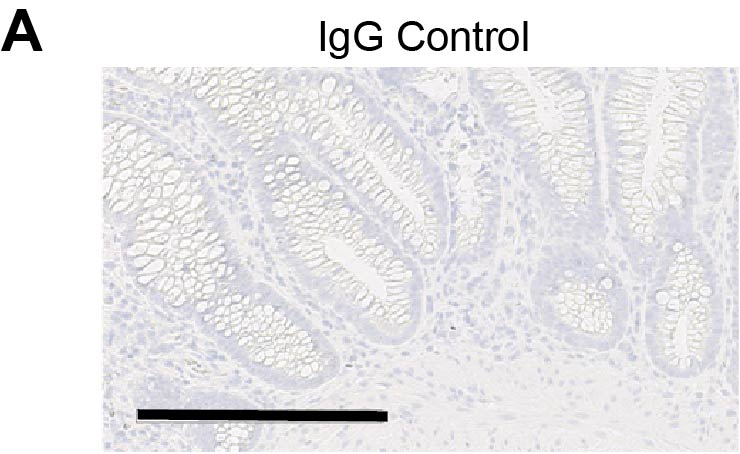

Supplement: jjaa054_suppl_Supplementary_Figure_7 [file jjaa054_suppl_supplementary_figure_7.jpeg]
