# Supplementary material for: Lymphocyte Activation Gene (LAG)-3 Is Associated With Mucosal Inflammation and Disease Activity in Ulcerative Colitis
Source: J Crohns Colitis. 2020 Mar 16;14(10):1446–61. doi: 10.1093/ecco-jcc/jjaa054 (PMC7533903; doi:10.1093/ecco-jcc/jjaa054)
Supplement: jjaa054_suppl_Supplementary_Table_1 [file jjaa054_suppl_supplementary_table_1.docx]

**Supplementary Table 1A - Antibody List**

| **Surface Marker** | **Fluorochrome** | **Clone** | **Cat No.** |
| --- | --- | --- | --- |
| **BD Biosciences** |  |  |  |
| CD3 | PE-CF594 | UCHT1 | 562310 |
| CD103 | BV711 | Ber-ACT8 | 563162 |
| CD25 | BV786 | M-A251 | 563701 |
| IL-4 | BV711 | MP4-25D2 | 564112 |
| **Biolegend** |  |  |  |
| CD4 | FITC | OKT4 | 317407 |
| Integrin β7 | FITC | FIB504 | 321213 |
| GM-CSF | PE-Dazzle | BVD2-21C11 | 502318 |
| IFNγ | PE-Dazzle | B27 | 506530 |
| CD25 | PE-Cy7 | BC96 | 302611 |
| CCR9 | PE-Cy7 | L053E8 | 358909 |
| CCR7 | APC | G043H7 | 353214 |
| CD45 | AF700 | HI30 | 304024 |
| γδTCR | BV421 | B1 | 331217 |
| TCRvα7.2 | BV421 | 3C10 | 351716 |
| Foxp3 | BV421 | 206D | 320124 |
| CD45RA | BV510 | HI100 | 304141 |
| CCR6 | BV605 | G034E3 | 353419 |
| CD8a | BV650 | RPA-T8 | 301041 |
| CXCR3 | BV711 | G025H7 | 353731 |
| CD127 | BV785 | A019D5 | 351330 |
| **eBioscience** |  |  |  |
| IL-17A | eFluor-450 | eBio64DEC17 | 48-7179 |
| Fixable Viability dye | eFluor-780 |  | 65-0865 |
| IL-10 | PE-Cy7 | JES3-9D7 | 25-7108-41 |
| IL-22 | PE-Cy7 | 22URTI | 25-7229 |
| **GSK** |  |  |  |
| LAG-3 | PE | J11L1 | 92540 |
| **Mitenyi Biotec** |  |  |  |
| CD161 | APC | 191B8 | 130-092-678 |

**Table 1B: TaqMan® Gene Expression Assays**

| **Gene** | **Assay ID** | **Company** |
| --- | --- | --- |
| *POLR2G* | Hs00275738_m1 | Thermofisher Scientific |
| *POLR2J* | Hs01558819_m1 | Thermofisher Scientific |
| *IL17A* | Hs00174383_m1 | Thermofisher Scientific |
| *IFNG* | Hs00989291_m1 | Thermofisher Scientific |
| *IL10* | Hs00961622_m1 | Thermofisher Scientific |

| **Gene** | **Assay ID** | **Forward Sequence** | **Reverse Sequence** | **Probe Sequence** | **Species** |
| --- | --- | --- | --- | --- | --- |
| *LAG3* Human Long | B100027 | CGCAGGCTCAGAGCAAGATA | ATTTGGACTGGGCTGCTGA | AGGAGCTGGAGCAAGAACCGGA | HUMAN |

**Table 1C: Immunofluorescence antibodies**

| **Antibody** | **Isotype** | **Dilution** | **Vendor** | **Cat No.** |
| --- | --- | --- | --- | --- |
| **CD4** | Rabbit IgG(SP35) | RTU (2.5µg/ml) | Ventana | 790-4423 |
| **CD8** | Rabbit IgG(SP57) | RTU | Ventana | 790-4460 |
| **LAG3** | Mouse IgG1(17B4) | 1:500 | LifeSpan Bio | LS-B2237 |

RTU; ready to use

**Table 1D: Immunohistochemistry antibodies**

| **Antibody** | **Isotype** | **Dilution** | **Vendor** | **Cat No.** |
| --- | --- | --- | --- | --- |
| **Calprotectin** | MAC 387 | 1:800 | Dako | ab22506 |
| **LAG3** | 17B4 | 1:500 | LifeSpan Bio | LS-C18692 |
