# Supplementary material for: Lymphocyte Activation Gene (LAG)-3 Is Associated With Mucosal Inflammation and Disease Activity in Ulcerative Colitis
Source: J Crohns Colitis. 2020 Mar 16;14(10):1446–61. doi: 10.1093/ecco-jcc/jjaa054 (PMC7533903; doi:10.1093/ecco-jcc/jjaa054)
Supplement: jjaa054_suppl_Supplementary_Table_2 [file jjaa054_suppl_supplementary_table_2.docx]

**Supplementary Table 2**. Patient information for single-cell RNA sequencing

|  | **UC**  **Inflamed** |
| --- | --- |
| Number | 4 |
| Age, median (range) | 35y (29-41) |
| M/F | 3/1 |
| **Disease Extent** |  |
| E1 Proctitis | 0 |
| E2 Left-sided  E3 Extensive | 3  1 |
| PSC  Disease duration,  median (range) | 0  7y (1-10) |
| UCEIS, median (range) | 4 (3-6) |
| **Medication at endoscopy** |  |
| Thiopurines | 1 |
| 5-ASA | 3 |
| Corticosteroids | 0 |
| Infliximab  Adalimumab | 0  1 |
| Vedolizumab | 0 |
