# Supplementary material for: Lymphocyte Activation Gene (LAG)-3 Is Associated With Mucosal Inflammation and Disease Activity in Ulcerative Colitis
Source: J Crohns Colitis. 2020 Mar 16;14(10):1446–61. doi: 10.1093/ecco-jcc/jjaa054 (PMC7533903; doi:10.1093/ecco-jcc/jjaa054)
Supplement: jjaa054_suppl_Supplementary_Table_3_A [file jjaa054_suppl_supplementary_table_3_a.docx]

**Supplementary Table 3A: T cell phenotype Panel 1**

| Panel 1 Phenotype | Subset  (as % of parent) | Avg % (± SEM) |
| --- | --- | --- |
| CD3 (as % of Live cells) |  | 30.03 ± 2.85 |
| CD4 | Naïve | 5.77 ± 0.86 |
|  | Effector | 1.84 ± 0.24 |
|  | Central Memory | 35.29 ± 3.44 |
|  | Effector Memory | 56.93 ± 3.54 |
|  | Regulatory | 4.97 ± 1.40 |
|  | Th1 | 24.08 ± 2.22 |
|  | Th0/2 | 38.02 ± 3.05 |
|  | Th17 | 23.33 ± 2.27 |
|  | Th1/Th17 | 13.95 ± 1.99 |
| CD8 | Naïve | 9.23 ± 1.92 |
|  | Effector | 12.36 ± 1.46 |
|  | Central Memory | 11.21 ± 2.20 |
|  | Effector Memory | 66.68 ± 3.61 |
| γδT cells (as % of CD3) |  | 1.90 ± 0.32 |
| CD3^-^ cells (as % of live cells) |  | 67.04 ± 3.01 |
