# Supplementary material for: Lymphocyte Activation Gene (LAG)-3 Is Associated With Mucosal Inflammation and Disease Activity in Ulcerative Colitis
Source: J Crohns Colitis. 2020 Mar 16;14(10):1446–61. doi: 10.1093/ecco-jcc/jjaa054 (PMC7533903; doi:10.1093/ecco-jcc/jjaa054)
Supplement: jjaa054_suppl_Supplementary_Table_3_B [file jjaa054_suppl_supplementary_table_3_b.docx]

**Supplementary Table 3B: T cell phenotype Panel 2**

| Panel 2 Phenotype | Subset  (as % of parent) | Avg % (± SEM) |
| --- | --- | --- |
| CD4 | CD161 | 54.97 ± 2.78 |
|  | CCR9^+^β7^-^ | 17.82 ± 2.57 |
|  | CCR9^-^ β7^+^ | 17.80 ± 3.57 |
|  | CCR9^+^β7^+^ | 9.87 ± 2.11 |
|  | CD103 | 7.93 ± 1.07 |
| CD8 | CD161 | 26.82 ± 2.92 |
|  | CCR9^+^β7^-^ | 8.43 ± 1.16 |
|  | CCR9^-^β7^+^ | 38.88 ± 3.05 |
|  | CCR9^+^β7^+^ | 16.87 ± 2.68 |
|  | CD103 | 45.48 ± 2.86 |
|  | MAITs | 2.98 ± 0.44 |
