# Supplementary material for: Lymphocyte Activation Gene (LAG)-3 Is Associated With Mucosal Inflammation and Disease Activity in Ulcerative Colitis
Source: J Crohns Colitis. 2020 Mar 16;14(10):1446–61. doi: 10.1093/ecco-jcc/jjaa054 (PMC7533903; doi:10.1093/ecco-jcc/jjaa054)
Supplement: jjaa054_suppl_Supplementary_Figure_Legends [file jjaa054_suppl_supplementary_figure_legends.docx]

# SUPPLEMENTARY FIGURE LEGENDS

**Figure 1**. **Novel approach to determine the upper UMI threshold for valid cell barcodes in single-cell RNA-sequencing data that utilises paired single-cell TCR-sequencing data.**

(**A-D**) Representative violin plots showing the log UMI count (A,C) or percent mitochondrial reads (B,D) for cells with zero, one, two or three TCRα (A,B) or TCRβ (C,D) chains. (**E**) Representative violin plot showing the UMI count for cells with one TCRα and/or one TCRβ chain. The upper UMI threshold (85^th^ percentile) is indicated by the red line. (**F,G**) Violin plots from (A) and (C) with upper (red) and lower (blue) UMI thresholds indicated. Wilcoxon Rank Sum test performed for the comparisons indicated. *** p < 0.001.

**Figure 2.** **LAG-3^+^ T cells are increased in the inflamed colon of patients with UC**

(**A**) Correlation of *LAG3* transcript from a subset of patients with UC (uninflamed and inflamed) with the Nancy score (n=24). ** p<0.01. Spearman correlation test was performed.

**Figure 3. LAG-3^+^ cells are enriched within the effector memory and central memory T cells populations**

(**A**) Simplified schematic and (**B-C**) full gating strategy used to identify the naïve, antigen experienced/memory and T helper subsets that are LAG-3^+^ (**Panel 1**). (**D**) Simplified schematic and (**E**) full gating strategy used to identify gut homing and mucosal-associated subsets that are LAG-3^+^ (**Panel 2**). The percentages of LAG-3 were identified on the T cell populations in black (**A** and **D**) and the populations in grey were part of the gating strategy. (**F**) t-SNE visualisation of mucosal-associated T cell clusters within the LAG-3^+^ cells of three UC patients with active disease.

**Figure 4.** ***LAG3*^+^ cells are a heterogeneous population of cytokine-expressing activated T cells**

**(A**) Transcriptionally distinct clusters of colonic CD3^+^ T cells from four UC patients with active disease, as visualised by UMAP. **(B)** Localisation of T cells from the four colon samples (one ascending and three distal) amongst the clusters shown in (A). **(C)** Bar chart showing the percent contribution of each sample to the 15 clusters of T cells shown in (A).

**Figure 5. *LAG3*^+^ cells are a heterogeneous population of cytokine-expressing activated T cells**

Pathway enrichment analysis of the genes upregulated within the CD4^+^ (**A**) and CD8^+^ (**B**) T cell clusters with the highest *LAG3* expression (CD4^+^ cluster 5 and CD8^+^ cluster 2).

**Figure 6.** **LAG-3^+^ colonic T cells predominantly secrete IFNγ and IL-17A**

(**A**) The levels of IL-10 produced by the CD4^+^CD25^+^FoxP3^+^ Tregs amongst the LAG-3^+^ and LAG-3^-^ T cells. (**B**) CD8^+^ LPMCs from patients with active UC (n=6) were extracted from colonic biopsies and stained for LAG-3, IFNγ, IL-17A and IL-10, with (red bars) and without (blue bars) PMA/Ionomycin stimulation. *p<0.05, **p<0.01. Comparisons for continuous data were performed using Mann-Whitney U test (**A**) and paired analyses with the Wilcoxon test (**B-C**).

**Figure 7. LAG-3 cell numbers correlate with endoscopic inflammation and are diminished in responders to biologic therapy**

(**A**) Representative IHC image of colonic mucosa stained for isotype control (IgG) from a patient with active UC.
